# Responding to ACL Injury and its Treatments: Comparative Gene Expression between Articular Cartilage and Synovium

**DOI:** 10.3390/bioengineering10050527

**Published:** 2023-04-26

**Authors:** Jonah I. Donnenfield, Benedikt L. Proffen, Braden C. Fleming, Martha M. Murray

**Affiliations:** 1 Division of Sports Medicine, Department of Orthopaedic Surgery, Boston Children’s Hospital, Harvard Medical School, Boston, MA 02115, USA; 2 Department of Orthopaedics, Warren Alpert Medical School of Brown University/Rhode Island Hospital, Providence, RI 02903, USA

**Keywords:** ACL, cartilage, synovium, PTOA, osteoarthritis, knee, reconstruction, RNA-seq

## Abstract

The relationship between cartilage and synovium is a rapidly growing area of osteoarthritis research. However, to the best of our knowledge, the relationships in gene expression between these two tissues have not been explored in mid-stage disease development. The current study compared the transcriptomes of these two tissues in a large animal model one year following posttraumatic osteoarthritis induction and multiple surgical treatment modalities. Thirty-six Yucatan minipigs underwent transection of the anterior cruciate ligament. Subjects were randomized to no further intervention, ligament reconstruction, or ligament repair augmented with an extracellular matrix (ECM) scaffold, followed by RNA sequencing of the articular cartilage and synovium at 52 weeks after harvest. Twelve intact contralateral knees served as controls. Across all treatment modalities, the primary difference in the transcriptomes was that the articular cartilage had greater upregulation of genes related to immune activation compared to the synovium—once baseline differences between cartilage and synovium were adjusted for. Oppositely, synovium featured greater upregulation of genes related to Wnt signaling compared to articular cartilage. After adjusting for expression differences between cartilage and synovium seen following ligament reconstruction, ligament repair with an ECM scaffold upregulated pathways related to ion homeostasis, tissue remodeling, and collagen catabolism in cartilage relative to synovium. These findings implicate inflammatory pathways within cartilage in the mid-stage development of posttraumatic osteoarthritis, independent of surgical treatment. Moreover, use of an ECM scaffold may exert a chondroprotective effect over gold-standard reconstruction through preferentially activating ion homeostatic and tissue remodeling pathways within cartilage.

## 1. Introduction

Osteoarthritis is one of the greatest contributors to physical disability in adults, and the growing burden of this disease continues to dwarf epidemiologic projections [1,2]. Unfortunately, there remains no disease-modifying treatment for osteoarthritis or its post-injury form—posttraumatic osteoarthritis (PTOA). PTOA has been shown to be strongly associated with injury to the anterior cruciate ligament (ACL), a condition that is also increasing in incidence [3]. These factors have motivated inquiries into the basic biology of PTOA, of which animal models have been particularly illuminating [4]. However, despite a plethora of multi-tissue and multi-omics animal studies, characterizing the pathogenesis of PTOA beyond 12 weeks after an initiating event remains underexplored [5,6,7,8,9,10].

We sought to fill this gap in the literature by comparing porcine transcriptomes between articular cartilage and synovium in the knee 52 weeks following ACL injury. We also sought to determine how cartilage and synovium differ in their transcriptomic responses to various forms of treating the torn ligament. We hypothesized that ACL injury (regardless of treatment) would induce inflammatory expression responses in both cartilage and synovium and that the response would be greater in synovium. We also posited that repairing the ligament with an extracellular matrix (ECM) scaffold would produce a significant decrease in the expression of these inflammatory pathways in synovium relative to cartilage, when adjusting for expression changes following gold-standard reconstruction, given that macroscopic cartilage damage following reconstruction has been shown to be greater than that following repair with an ECM scaffold [11,12].

## 2. Materials and Methods

### 2.1. Study Design

Thirty-six adolescent Yucatan minipigs (Sinclair BioResources, Columbia, MO, USA) were included in this study. Approval from the Brown University Institutional Animal Care and Use Committee was obtained prior to the study (Protocol number: 1511000175), which was designed following the ARRIVE guidelines [13]. The gait metrics and cartilage integrity for these animals have been previously reported [11]. All 36 Yucatan minipigs were allocated to undergo unilateral ACL transection (n = 36) followed by no further treatment (ACLT, n = 12), reconstruction (RECON, n = 12), or surgical repair with an ECM scaffold (REPAIR, n = 12) of the ligament. Euthanasia was performed at 52 weeks after surgery. To generate control samples, sex-, surgical-group-, and knee-laterality-stratified randomization selected 4 contralateral knees from each surgical group to establish 12 control samples (CON, n = 12) to which surgical joints would be compared. After surgery, investigators were blinded to animal group assignments for all outcome assessments. Justification for the Yucatan minipig model and details on the IACUC-approved surgical procedures, animal husbandry, and pain management can be found in Supplement S1 along with the IACUC approval letter.

### 2.2. Extraction of Articular Cartilage and Synovium

After euthanasia, osteochondral samples were extracted from the medial femoral condyle—with RNA isolation samples coming from the surface posterior to the frontal plane at the center of the medial femoral condyle. In total, Four to eight 5 mm osteochondral biopsies were taken from each animal, and these samples were subsequently rinsed with water and separated from the attached bone. They were then flash-frozen in liquid nitrogen and placed in a −80 °C freezer until homogenization and RNA isolation. To extract synovium samples, the medial meniscus of the surgical leg along with the attached synovium and capsule were excised. A sample of synovium posterior to the frontal plane through the center of the pars intermedia was harvested, immediately homogenized, and then flash-frozen for later RNA isolation as described below.

### 2.3. Articular Cartilage and Synovium RNA-Seq

To process cartilage tissues, the specimens were homogenized in 2 mL tubes (MP Biomedical, Santa Ana, CA, USA) containing 500 μL of frozen TRIzol (Life Technologies, Carlsbad, CA, USA) using a sterile drill bit while tubes were submerged in liquid nitrogen. After one round of drilling, tubes received an additional 500 μL of liquid TRIzol, were flash-frozen in liquid nitrogen, and were again homogenized using the drill bit. Total RNA was extracted using phenol-chloroform and purified using PureLink RNA Mini Kit (Life Technologies). Samples were then checked for purity with NanoDrop (Thermo Scientific, Cambridge, MA, USA) and checked for integrity using a combination of Agilent Tapestation High Sensitivity RNA Screen Tape and Agilent Tapestation RNA Screen Tape. Mean 260/280 absorbance ratios were 1.7 and 1.8 for controls and surgical groups, respectively (Table 1). Mean 260/230 ratios were 1.2 and 1.5, and mean RIN integrity numbers (RINs) were 6.1 and 5.6, respectively (Table 1).

To process synovium tissues, the specimens were immediately placed in 2 mL lysing matrix S tubes (MP Biomedicals) and homogenized in 1 mL TRIzol (Life Technologies) using a Fast Prep-24 Instrument (MP Biomedical). Samples were then flash-frozen in liquid nitrogen, transferred to dry ice, and stored at −80 °C until RNA isolation. Total RNA was extracted using phenol-chloroform and purified using PureLink RNA Mini Kit (Life Technologies). Samples were checked for purity with NanoDrop (Thermo Scientific) and checked for integrity using a combination of Agilent Tapestation High Sensitivity RNA Screen Tape and Agilent Tapestation RNA Screen Tape. Mean 260/280 absorbance ratios were 2.0 and 2.0 for controls and surgical groups, respectively (Table 1). Mean 260/230 ratios were 1.9 and 1.9, and mean RIN integrity numbers (RINs) were 6.2 and 5.5, respectively (Table 1).

All cartilage and synovium RNA isolates were then library-prepped using KAPA mRNA HyperPrep with RiboErase (Roche, Basel, Switzerland) and subsequently pooled and sequenced together on a NovaSeq 6000 S2 Flow Cell with 100 bp paired-end reads (Biopolymers Facility, Harvard Medical School, Boston, MA, USA). Fastq files for cartilage and synovium samples were piped into FastQC version 0.11.9 to create individual sample reports that were then compiled using MultiQC version 1.12 [14,15]. Summary statistics and histograms of mean quality values across each base position of the read were generated (Supplement S2—MultiQC Reports). All cartilage samples were contained within a Phred score range of 31.80 to 39.36. All synovium samples were contained within a Phred score range of 25.05 to 38.84. Using Salmon version 1.8.0, reads were quasi-mapped, and transcript quantification files were generated [16]. Mapping employed the Sscrofa11.1 porcine genome, which was assembled by The Swine Genome Sequencing Consortium (SGSC) and hosted by Ensembl (http://ftp.ensembl.org/pub/current_embl/sus_scrofa/, accessed on 20 February 2023).

For surgical samples, the mean numbers (and %) of uniquely mapped reads were 31.0 million (78.4%) and 28.7 million (77.2%) for cartilage and synovium, respectively (Table 1). For control samples, the mean numbers (and %) of uniquely mapped reads were 29.7 million (78.2%) and 29.0 million (81.7%) for cartilage and synovium, respectively (Table 1).

### 2.4. Statistical Analysis

Demographic, RNA quality, and sequencing characteristics for surgical subjects and contralateral controls were analyzed in R version 4.2.1 with Mann–Whitney tests and Fisher exact tests because visual inspection revealed non-normal distributions [17]. R output for these calculations, along with summary statistics calculations and raw demographic data, can be found in Supplement S3.

### 2.5. Differential Gene Expression Analysis

**Figure 1:**
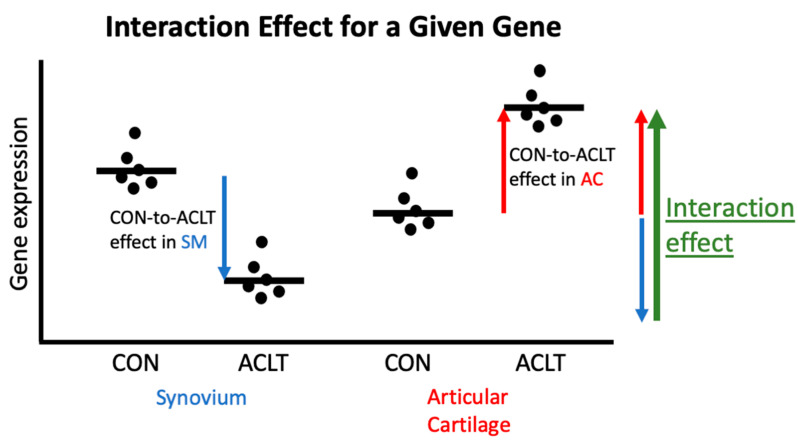
Visualization of the interaction effect calculation for each gene. For each tissue type, expression in control (CON) samples is subtracted from expression in ACLT samples. This creates the treatment effects in synovium (blue arrow) and articular cartilage (red arrow), respectively. The treatment effect in synovium is then subtracted from the treatment effect in cartilage to calculate the interaction effect (green arrow). The magnitude and direction of the interaction effect (provided by a L2FC) represent how the treatment (e.g., ACLT) differs between cartilage and synovium when controlling for a baseline effect (e.g., CON). In the provided example, a positive interaction effect means ACLT induced more upregulation of this gene in articular cartilage than it did in synovium after adjusting for differences in CON expression between the two tissues.

Differential gene expression analysis was performed in R version 4.2.1 using DESeq2 with RUVSeq adjustment on transcript quantification files produced by Salmon mapping [17,18,19]. *p*-values were adjusted for multiple testing using the Benjamini–Hochberg false discovery rate (FDR) with a value of <0.05 as the cutoff for inclusion. Principal component analysis (PCA) plots of differentially expressed genes were used for high-level visualization of samples, and outliers were removed according to stark separation in first four principal components (Supplement S4—PCA outliers). To reduce the noise of baseline differences between cartilage and synovium transcriptomes, expression analysis focused on calculating the interaction effect between tissue type and treatment type. For each comparison between cartilage and synovium, one form of treatment (i.e., ACLT) served as the treatment of focus while another (i.e., CON) served as a baseline to be subtracted (Figure 1). This resulted in log2 fold change (L2FC) values for the interaction effects. To ensure tissue comparisons were not driven by a single sample, each gene was only considered differentially expressed if at least two samples provided non-zero expression values.

### 2.6. Functional Pathway Analysis

Overrepresentation analysis used hypergeometric testing on the differentially expressed gene lists and tested for representation of Gene Ontology (GO) terms [20]. These terms included biological processes, molecular functions, and cellular components, and the priority of reporting these terms was assigned in that order (i.e., if biological process and molecular function terms were both present, biological processes were preferentially documented). Clusterprofiler created category netplots of GO terms, and Revigo treemaps grouped GO terms by parent terms for high-level visualization [21,22]. Category netplots were used for comparisons where fewer GO terms were present, and there was an emphasis on showcasing influential genes. Treemaps were used for comparisons where more GO terms were present, and there was an emphasis on high-level visualization. Gene Set Enrichment Analysis (GSEA) was performed to assess Kyoto Encyclopedia of Genes and Genomes (KEGG) pathway enrichment [23,24]. An adjusted *p*-value of <0.05 was used for all pathway analysis methods to provide a cutoff for term inclusion.

## 3. Results

Baseline age, weight, and sex did not differ among surgical subjects or their contralateral controls for either tissue type (Table 1).

### 3.1. Differential Gene Expression Analysis by Experimental Group

Articular cartilage and synovium featured 329, 1210, and 330 differentially expressed genes when compared within ACLT, RECON, and REPAIR subjects, respectively, after adjusting for cartilage vs. synovium differences in CON samples (Table 2). Furthermore, 97 and 64 genes were differentially expressed within RECON and REPAIR samples, respectively, when baseline cartilage vs. synovium differences in ACLT samples were adjusted for (Table 2). Ninety-nine genes were differentially expressed within REPAIR samples when baseline cartilage vs. synovium differences in RECON samples were adjusted for (Table 2). When all 3 treatment groups were pooled and cartilage was compared to synovium in the treated knees, there were 1227 differentially expressed genes after adjusting for cartilage vs. synovium differences in CON samples (Table 2).

### 3.2. Functional Pathway Analysis with Control Samples as Baseline

**Figure 2:**
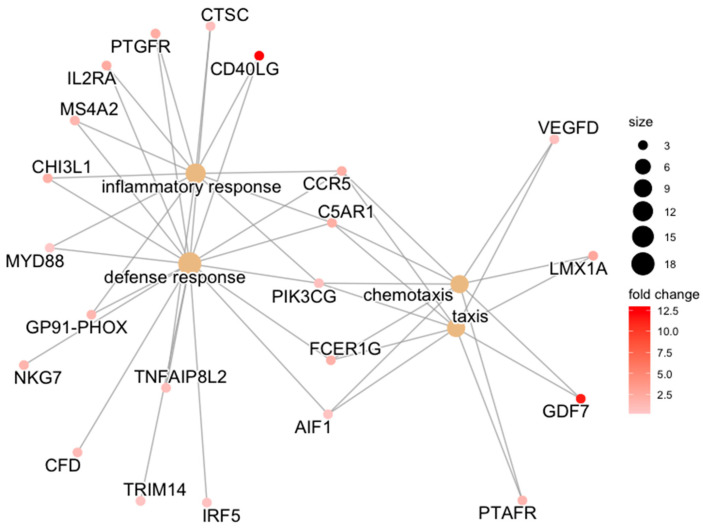
Category netplot of biological process GO terms. Category netplot of biological process GO terms and their constituent upregulated genes, which were overrepresented among the list of 329 differentially expressed genes between cartilage and synovium for the ACLT comparison, adjusting for CON differences between tissues. GO term size is proportional to how many genes contribute to it, and L2FC of gene expression between tissues is color-coded.

Controlling for baseline differences in CON expression between articular cartilage and synovium, ACLT articular cartilage samples featured greater upregulation of the biological processes defense response, inflammatory response, and chemotaxis (Table 3, Figure 2) compared to the ACLT synovium samples.

**Figure 3:**
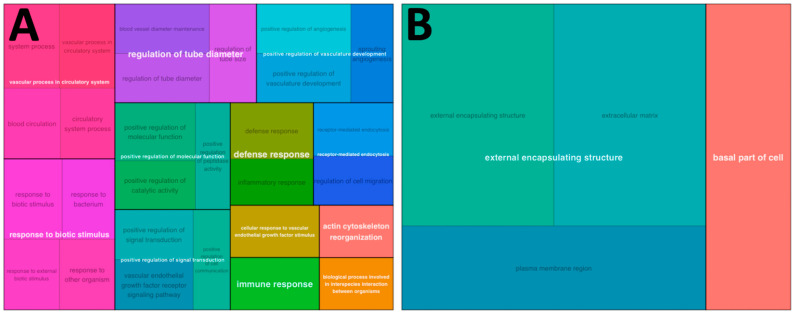
Treemap plots of upregulated and downregulated GO terms. Treemap plots of (**A**) upregulated biological process GO terms and (**B**) downregulated cellular component GO terms between RECON cartilage samples and RECON synovium samples, adjusting for baseline CON differences. GO terms are grouped and colored according to unifying parent terms, and the amount of space a term occupies is proportional to gene set size and hypergeometric testing of overrepresented genes.

After the same baseline CON adjustment, RECON cartilage samples expressed upregulation of biological processes related to angiogenesis (e.g., vascular process in circulatory system) and immune activation (e.g., defense response, immune response) relative to RECON synovium samples (Table 3, Figure 3A). There was also downregulation of the cellular component extracellular matrix in cartilage relative to synovium (Figure 3B).

**Figure 4:**
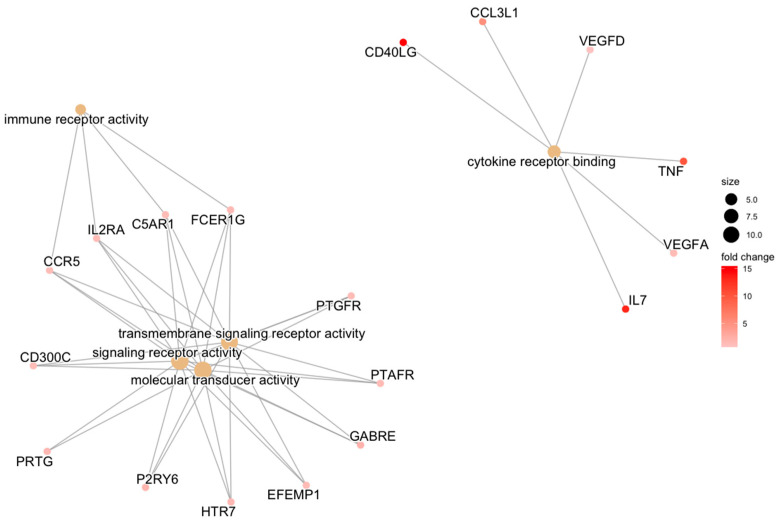
Category netplot of molecular function GO terms. Category netplot of molecular function GO terms and their constituent upregulated genes, which were overrepresented among the list of 330 differentially expressed genes between cartilage and synovium for the REPAIR comparison, adjusting for CON differences between tissues. GO term size is proportional to how many genes contribute to it, and L2FC of gene expression between tissues is color-coded.

REPAIR cartilage samples also featured relative upregulation of immune pathways relative to REPAIR synovium samples after adjusting for CON differences. This was represented by molecular functions such as cytokine receptor binding (driven by *CD40LG*, *TNF*, and *IL7*) and immune receptor activity (driven by *CCR5*, *IL2A*, and *C5AR1*) (Table 4, Figure 4).

**Figure 5:**
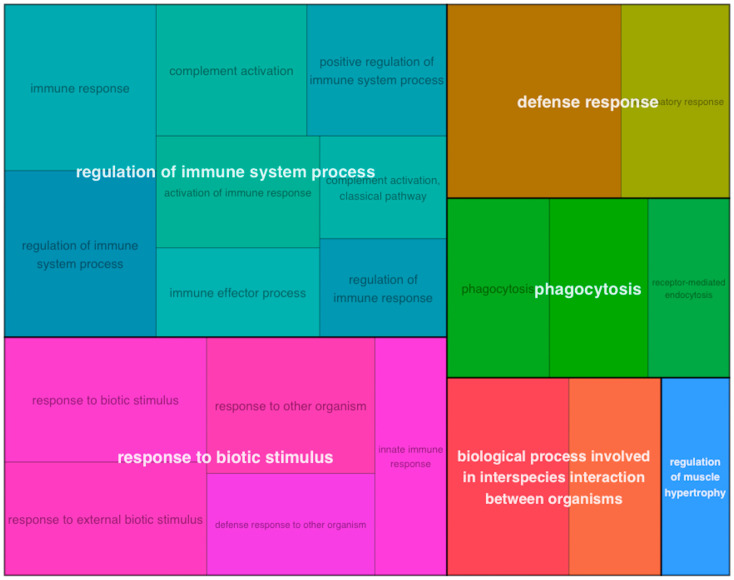
Treemap plot of upregulated biological process GO terms. Treemap plot of upregulated biological process GO terms for comparing pooled cartilage samples to pooled synovium samples, adjusting for baseline CON differences. GO terms are grouped and colored according to unifying parent terms, and the amount of space a term occupies is proportional to gene set size and hypergeometric testing of overrepresented genes.

**Figure 6:**
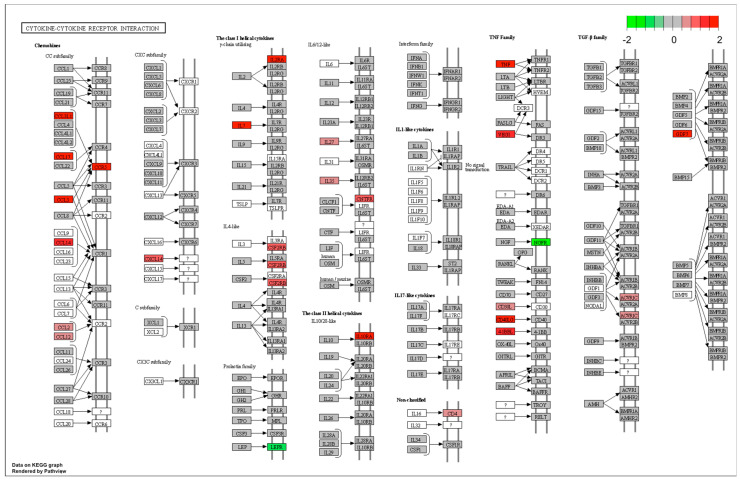
Pathview diagram of the KEGG pathway *cytokine*–*cytokine receptor interaction*. Pathview diagram of the KEGG pathway *cytokine*–*cytokine receptor interaction* following Gene Set Enrichment Analysis of pooled cartilage samples vs. pooled synovium samples, adjusting for baseline CON differences between the two groups. Rectangles represent genes. Positive L2FC (i.e., upregulated in cartilage relative to synovium) is represented by red, and negative L2FC (i.e., downregulated in cartilage relative to synovium) is represented by green. Genes in white did not have L2FC values.

**Figure 7:**
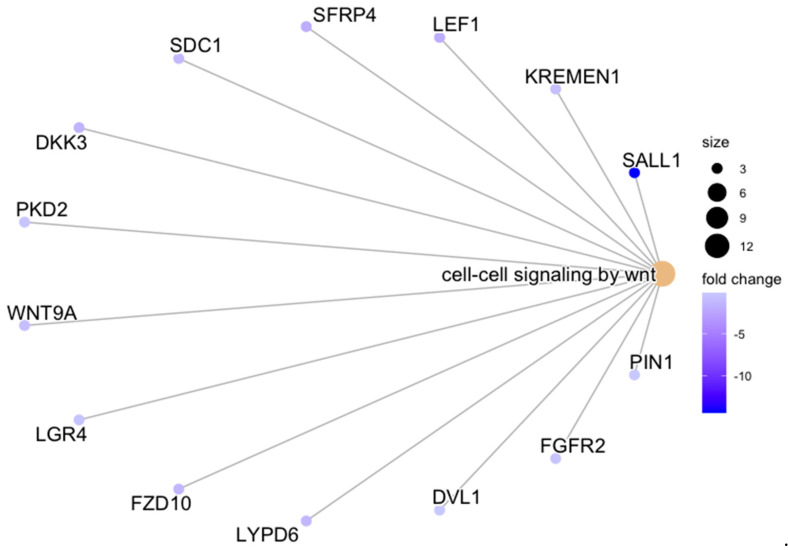
Category netplot of the biological process GO term *cell*–*cell signaling by wnt*. Category netplot of the biological process GO term *cell*–*cell signaling by wnt* and its constituent downregulated genes, which were overrepresented among the list of 1227 differentially expressed genes between cartilage and synovium for the pooled comparison, adjusting for CON differences between tissues. GO term size is proportional to how many genes contribute to it, and L2FC of gene expression is color-coded.

**Figure 8:**
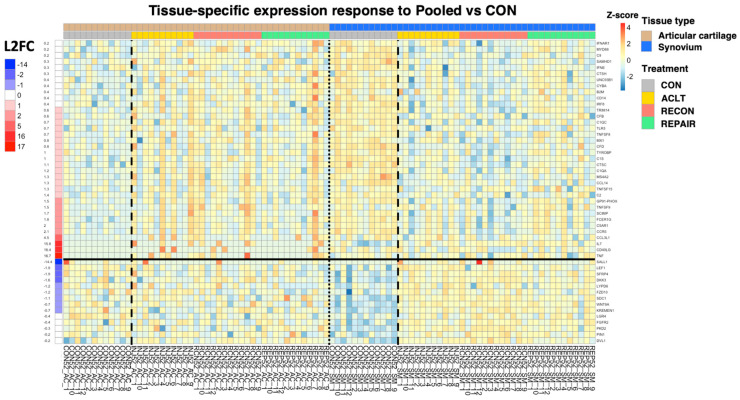
Heatmap of expression for genes comprising biological process GO terms *immune response* and *cell*–*cell signaling by wnt*. Heatmap of expression for genes comprising biological process GO terms *immune response* (rows above solid black line) and *cell*–*cell signaling by wnt* (rows below solid black line) for articular cartilage vs. synovium pooled treatment groups, adjusting for baseline CON differences between tissues. Dashed lines separate CON samples from treatment samples. The dotted line separates cartilage from synovium. Tissue type and treatment groups are indicated by column headers, gene names are labeled to the right of their respective rows, L2FCs are labeled to the left of rows and are color-coded, and sample IDs are labeled below their respective columns. Z-scores were calculated independently for articular cartilage and synovium.

When all three treatment groups were pooled together (i.e., ACLT, RECON, and REPAIR were combined) and compared between tissues—adjusting for CON differences—biological processes related to immune activation were upregulated in cartilage relative to synovium (Figure 5). These terms included *immune response*, *complement activation*, and *positive regulation of immune system process* (Figure 5). GSEA of KEGG pathways revealed upregulation of *cytokine*–*cytokine receptor interaction*, which was influenced by increased cartilage expression of several chemokines and members of the TNF family (Figure 6). Notably, there was upregulation of the biological process *cell*–*cell signaling by wnt* in synovium relative to cartilage after adjusting for baseline CON differences (Figure 7 and Figure 8).

### 3.3. Functional Pathway Analysis with Experimental Groups as Baseline

Controlling for baseline differences in ACLT expression between articular cartilage and synovium, RECON articular cartilage samples featured greater downregulation of molecular functions related to vitamin B6 (e.g., pyridoxal phosphate binding and vitamin B6 binding) compared to RECON synovium samples. Overrepresentation analysis of differentially expressed genes between REPAIR cartilage samples and REPAIR synovium samples did not reveal enrichment of any GO terms.

**Figure 9:**
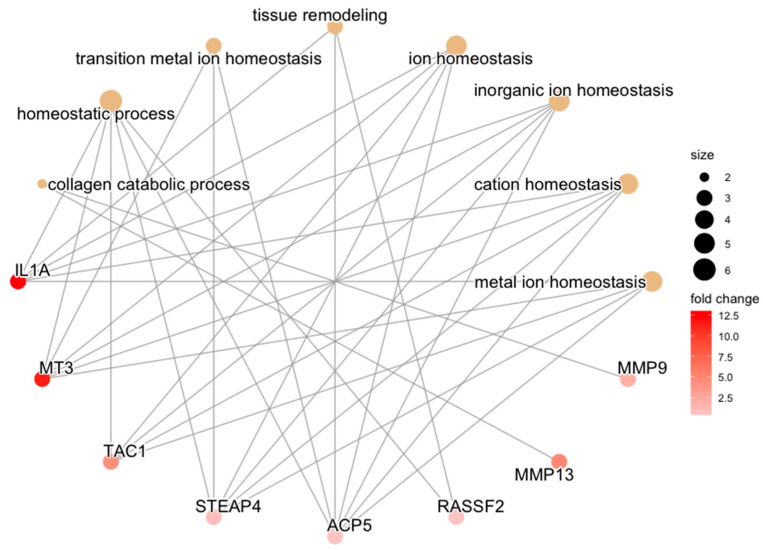
Category netplot of biological process GO terms. Category netplot of biological process GO terms and their constituent upregulated genes, which were overrepresented among the list of 99 differentially expressed genes between cartilage and synovium for the REPAIR comparison, adjusting for RECON differences between tissues. GO term size is proportional to how many genes contribute to it, and L2FC of gene expression is color-coded.

**Figure 10:**
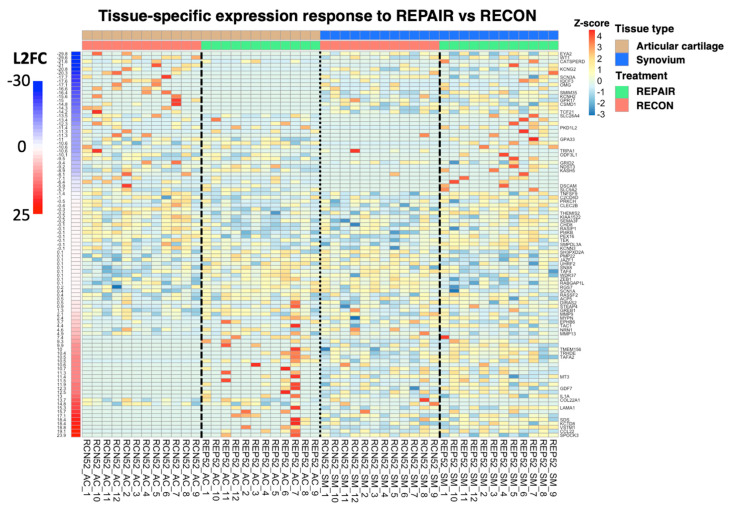
Heatmap of expression for the 99 differentially expressed genes between articular cartilage and synovium. Heatmap of expression for the 99 differentially expressed genes between articular cartilage and synovium following REPAIR, adjusting for baseline differences in RECON between tissues. Dashed lines separate RECON samples from REPAIR samples. The dotted line separates cartilage from synovium. Tissue type and treatment groups are indicated by column headers, gene names are labeled to the right of their respective rows, L2FCs are labeled to the left of rows and are color−coded, and sample IDs are labeled below their respective columns. Z−scores were calculated independently for articular cartilage and synovium. Rows missing gene names represent novel Sus scrofa genes or those with other-species orthologues.

Controlling for baseline differences in RECON expression between articular cartilage and synovium, REPAIR cartilage samples featured upregulation of biological processes related to ion homeostasis, tissue remodeling, and collagen catabolism (Figure 9 and Figure 10).

## 4. Discussion

The current study investigated the relationship between cartilage and synovium gene expression during the mid-stage development of PTOA. Differential gene expression analysis after ACL transection showed how transcriptomes between these two tissues largely differed in their expression of genes related to immune activation once baseline control differences were adjusted for—with cartilage having greater upregulation of immune-activation-associated genes than synovium. While this confirmed our prediction that immune/inflammatory pathways would be differentially regulated between cartilage and synovium at 52 weeks, the direction of the effect between the tissues was contrary to our hypothesis (i.e., ACL injury induced a greater immune response in cartilage than it did in synovium). There was also unanticipated differential regulation between tissues of canonical and non-canonical Wnt signaling, for which synovium featured greater upregulation than articular cartilage. Also contrary to our prediction, ligament repair did not produce a significant difference in the expression of immune-related pathways between cartilage and synovium. Instead, after adjusting for tissue expression differences seen in ligament reconstruction, repairing the ligament with an ECM scaffold upregulated pathways related to ion homeostasis, tissue remodeling, and collagen catabolism in cartilage relative to synovium.

The relationships between tissues, such as that between articular cartilage and subchondral bone, have played a fundamental role in understanding the development of osteoarthritis, and the interplay of cartilage and synovium is a rapidly growing area of osteoarthritis research [25]. Studies characterizing both cartilage and synovium in end-stage disease have identified a spectrum of inflammatory subtypes—with more damaged cartilage (as opposed to synovium) being associated with greater activation of immune pathways and extracellular matrix (ECM) remodeling [26,27,28,29]. Other evidence suggests that synovium (as opposed to cartilage) immune activation is the more prominent contributor to joint inflammation [30]. By identifying upregulation of inflammatory and immune pathways in cartilage relative to synovium after controlling for differences between control samples, the current study affirms the inflammatory role of cartilage and its contribution to disease progression even 52 weeks following ACL injury. Moreover, because the current study controlled for baseline differences in cartilage and synovium gene expression, these pathway findings are more reliably attributable to joint disease and not just constitutive differences between cartilage and synovium.

However, immune activation can take several forms, and not all forms of inflammation are equivalent. Diversity of immune function is well characterized in wound healing in tissues such as skin and lung, but far less is known about the diversity of immune mechanisms that regulate healing in load-bearing musculoskeletal tissues after injury [31,32,33]. The current study showed that following every form of surgical intervention, the cartilage responded with greater immune activation than the synovium in mid-term osteoarthritis after adjusting for baseline differences between the tissues. Interestingly, surgical groups featured similar expression patterns across the 36 genes that comprise the immune response GO term (Figure 8), which suggests leaving the ACL transected, reconstructing it, or repairing it with an ECM scaffold induces a similar pattern of immune-related gene expression in the articular cartilage. This is further supported by similarities in immune-related GO terms that resulted from unsupervised overrepresentation analysis; biological processes in both ACLT and RECON samples heavily depended on upregulation of *CD40LG*, *FCER1G*, *C5AR1*, *CCR5*, and *GP91-PHOX* (Table 3), and molecular functions (e.g., immune receptor activity) in RECON and REPAIR similarly depended on upregulation of *FCER1G*, *C5AR1*, *CCR5*, and *IL2RA* (Table 4). The current study advances our understanding of the pathogenesis of PTOA by suggesting that similar inflammatory phenotypes may be present even one year after ACL injury, regardless of surgical treatment.

There is ample evidence that canonical, β-catenin-dependent Wnt signaling in chondrocytes is associated with cartilage damage when constituent genes are under- or over-expressed [34,35,36]. Increased canonical Wnt signaling in synovium is also thought to contribute to cartilage damage through increased production of matrix metalloproteinases (MMPs) in synovium [37]. The current study affirms this thought by showing synovial upregulation of genes related to canonical Wnt signaling (e.g., *SALL1*, *LEF1*, *FZD10*, *DKK3*) in ACL-injured joints relative to controls (Figure 8). However, the role of non-canonical, β-catenin-independent Wnt signaling in both cartilage and synovium in the development of osteoarthritis is much less certain. GWAS of hand osteoarthritis has identified non-canonical expression of *WNT9A* in cartilage as associated with disease development, and experimental attempts to link non-canonical Wnt signaling in cartilage to osteoarthritis are ongoing [38,39]. In synovium, the relationship between non-canonical Wnt signaling and osteoarthritis development has been minimally explored [39]. The current study provides a robust contribution to this gap in the literature by associating upregulation of non-canonical Wnt signaling in synovium with mid-term PTOA. As Figure 8 shows, expression of *WNT9A* (which encodes a non-canonical Wnt ligand) and *LGR4* (which encodes a mediator of non-canonical Wnt-PCP signaling) is downregulated (i.e., shaded blue) in the synovium of uninjured joints and upregulated (i.e., shaded yellow/red) in the synovium of injured joints at one year after injury [40]. There are minimal discrepancies in the expression of Wnt-related genes in the cartilage from control joints relative to cartilage from injured joints. These findings strongly suggest an increase in both canonical and non-canonical Wnt signaling in the synovium is associated with posttraumatic osteoarthritis development 52 weeks following ACL injury, and gene expression in cartilage is relatively non-contributive at this timepoint.

Beyond modeling the joint response to ACL injury, previous animal models of ACL transection followed by ligament reconstruction have featured RNA sequencing of several knee joint tissues (e.g., bone, tendon, cartilage, synovium) [33,41,42]. Sieker et al. 2018 characterized cartilage and synovium transcriptomes in a porcine model at one and four weeks following ACL transection, with and without reconstruction [41,42]. In the setting of no macro- or microscopic differences in cartilage damage or synovitis across treatment groups, gene expression of all surgical subjects pooled together featured upregulation of pathways related to immune response and inflammation in cartilage and synovium at those early timepoints [41,42]. However, pathway comparisons were neither made between treatment modalities nor made between tissue types. Therefore, the current study, to the best of our knowledge, is one of the first to compare tissue transcriptomes between surgical treatments for ACL reconstruction and between cartilage and synovium. Moreover, it may be the only study to date to compare cartilage and synovium transcription profiles at a mid-stage timepoint in PTOA development, as most studies evaluate expression in early- or end-stage disease [28,43,44,45].

When cartilage and synovium samples from REPAIR subjects were compared, and tissue differences following gold-standard reconstruction (i.e., RECON) were subtracted out, the current study effectively isolated the net effect of ligament repair with an ECM scaffold on the transcriptomic relationship between cartilage and synovium. Ligament repair with an ECM scaffold developed by Murray et al. in 2013 has previously been shown to confer chondroprotection in a 52-week PTOA porcine model, and similar results were reproduced for the RECON and REPAIR subjects used in the current study [11,12]. Significant transcriptomic differences in the interaction effect between tissue types and REPAIR vs. RECON in this study suggest that gene expression may play a role in the outcomes produced by these two procedures. Upregulation of ion homeostasis pathways in cartilage relative to synovium was unique to REPAIR subjects and was not featured in RECON subjects (Figure 9). Previous studies have found ion homeostasis in chondrocytes to be closely linked to the pericellular environment—in terms of both extracellular matrix composition and oxygen tension [46]. Yuan et al. 2021 identified expression pathways specifically related to dysregulation in calcium ion homeostasis to associate with cartilage degeneration in end-stage disease samples [45]. However, the pooling of cartilage, subchondral bone, and synovium for that analysis left the tissue-specific origin of these expression patterns unresolved [45]. The current study revealed genes (e.g., *MT3*) that encode proteins that regulate divalent cation interactions to be comparatively upregulated in cartilage relative to synovium in REPAIR subjects [47]. This is similar to an early-stage canine study that found perturbation of voltage-gated Ca^2+^ channels with a small molecule inhibitor mitigated cartilage catabolism [48]. These findings suggest that affecting the ionic, possibly calcium-related, milieu within chondrocytes by repairing the ligament (as opposed to reconstructing the ligament) may be central to modulating cartilage degeneration up to one year following joint injury.

MMPs related to collagen breakdown (e.g., *MMP9* and *MMP13*) were also upregulated in the cartilage of REPAIR subjects relative to synovium at 52 weeks following ACL injury. These MMPs (and several others) have been identified in synovial fluid following ACL injury and have been noted to be produced by both chondrocytes and synoviocytes [49,50,51]. However, there is minimal documentation on the comparative production of MMPs between cartilage and synovium following ACL injury. In reconstructed and non-reconstructed joints alike, MMP-13 (encoded by *MMP13*) may be dominantly contributed by synovium in the first few weeks following ACL injury, but—to the best of our knowledge—no study has characterized the tissue-specific secretion of this type II collagen-degrading enzyme in the months following ACL injury [52,53]. When studied in isolation, chondrocytes upregulate *MMP13* expression following stimulation by IL-1β, but that has been documented in highly controlled environments and not within in vivo ACL injury animal models [54]. The current study, therefore, makes several contributions to the understanding of *MMP13* expression in the development of osteoarthritis. Firstly, one year following ACL injury, this MMP continues to be expressed by both cartilage and synovium. Secondly, repairing the ligament preferentially upregulates *MMP13* in cartilage relative to synovium, after adjusting for tissue differences following ligament reconstruction. That is, repairing the ligament may increase the relative expression of *MMP13* between cartilage and synovium, while reconstructing the ligament does not. However, chondrocyte upregulation of MMPs associated with cartilage breakdown seems antithetical to the diminished PTOA benefit that has been documented in this porcine model following REPAIR [11,12]. One explanation for this apparent paradox may be that RECON subjects also featured upregulation of these MMPs in cartilage but at an earlier timepoint; the same PTOA pathways may be activated in both RECON and REPAIR tissues, but this may be happening in a delayed fashion in REPAIR subjects relative to RECON subjects.

The current study has several limitations. Cartilage and synovium from contralateral joints served as controls instead of tissue from surgery-naïve subjects. The motivation for choosing this control paradigm was three-fold: (1) A prior large animal study showed that contralateral joints remain healthy four and a half years after unilateral ACL transection, though minor differences in the cartilage of the contralateral joint have been noted in a previous 12-month porcine model when comparing ligament repair to ligament reconstruction [12,55]. (2) The marginal benefit of using surgery-naïve subjects versus contralateral tissue was disproportionate to the financial and humane costs of acquiring and maintaining 12 additional experimental subjects for 12 months. (3) The study was conducted to minimize the number of animal subjects. An added benefit of using intact contralateral joints was that it minimized between-subject variance. Another limitation of the current study is that neither control animals nor contralateral knees underwent sham surgery. Therefore, joint bleeding and other healing processes may also have contributed to differential gene expression results seen in the surgical knees. However, joint harvest took place 52 weeks following surgery, so any post-surgical processes should have resolved by this time. The sample size of the current study was a sample of convenience, as the study was initially designed to evaluate gait changes and cartilage integrity in a prior analysis [11]. Cartilage samples were flash-frozen following harvest and subsequently thawed during homogenization and RNA isolation. Synovium samples were flash-frozen after an initial round of homogenization that took place at the time of tissue harvest. Synovium was then thawed for RNA isolation and flash-frozen again. Thus, both cartilage and synovium underwent multiple freeze–thaw cycles, which may have negatively impacted the quality of RNA. To account for this, RNA isolate samples were run on Agilent Tapestation RNA Screen Tape immediately before sequencing (as described in the Methods section), so RIN values are representative of final RNA integrity. As seen in Figure 10, some of the differentially expressed genes with the largest log2 fold changes were influenced by a small number of subjects. Therefore, our bioinformatics analysis required that at least two samples contribute to the featured signal for the gene to be considered, so there are no instances where L2FC is dependent on only one sample. Lastly, OARSI cartilage scoring guidelines were designed for goats and sheep but were extended to pigs, as previously done [11,12,42].

The current study makes several novel contributions to understanding the development of PTOA. One year following ACL transection, there was greater upregulation in the gene expression of immune response pathways in the cartilage when compared to the synovium, regardless of whether the ligament was reconstructed, repaired, or left untreated. Oppositely, synovium upregulated expression of genes related to canonical and non-canonical Wnt signaling relative to articular cartilage—also regardless of post-injury treatment modality. Importantly, transcriptomic heterogeneity in these pathways between cartilage and synovium became apparent after adjusting for baseline differences between cartilage and synovium control tissues, suggesting that the transcriptomic differences are due to tissue-specific responses to joint trauma and not simply due to differences in constitutive gene expression. The current study also showed that repairing the ACL with an ECM scaffold after transection is associated with distinct transcription responses in cartilage and synovium that are not present after reconstructing the ACL. These responses, related to ion homeostasis, tissue remodeling, and collagen catabolism, were upregulated in cartilage relative to synovium one year following injury and treatment. The findings depict cartilage as having a more reactionary inflammation/immune response to injury than synovium one year following injury. Moreover, surgical repair with an ECM scaffold may exert its chondroprotective effect through pathways related to ion homeostasis and tissue remodeling within articular cartilage.

## Figures and Tables

**Table 1:** RNA quality and sequencing characteristics. RNA quality and sequencing characteristics for all surgical samples combined (Pooled) and controls (CON) for articular cartilage (AC) samples and synovium (SM). Ideal 260/280 and 260/230 absorbance ratios are approximately 2.0. RNA integrity numbers (RIN) above 5 are acceptable for samples having undergone rRNA-depleted library prep.

	AC CON	AC Pooled	AC Pooled/CON	SM CON	SM Pooled	SM Pooled/CON
	Mean (Range)	Mean (Range)	*p*-value (95% CI)	Mean (Range)	Mean (Range)	*p*-value (95% CI)
Demographics						
Age (mo)	15 (13, 17)	15.3 (13, 18)	0.42 (−1.0, 1.0) ^a^	15 (13, 17)	15.3 (13, 18)	0.42 (−1.0, 1.0) ^a^
Weight (kg)	50.8 (40, 60)	51.9 (40, 60)	0.65 (−5.0, 2.0) ^a^	50.8 (40, 60)	51.9 (40, 60)	0.65 (−5.0, 2.0) ^a^
Sex (prop. female)	0.5	0.5	1 ^b^	0.5	0.5	1 ^b^
RNA quality						
Conc. (ng/µL)	17.2 (1.6, 33.8)	26.0 (7.5, 53.2)	0.05 (−16.7, 0.5) ^a^	112.9 (31.6, 377)	146.8 (14.1, 558)	0.31 (−86.1, 24.8) ^a^
260/280	1.7 (1.5, 1.9)	1.8 (1.6, 1.9)	0.03 (−0.2, −0.01) ^a^	2.0 (1.9, 2.1)	2.0 (1.8, 2.1)	0.95 (−0.02, 0.03) ^a^
260/230	1.2 (0.4, 1.8)	1.5 (1.6, 1.9)	0.17 (−0.5, 0.06) ^a^	1.9 (1.5, 2.2)	1.9 (0.6, 2.2)	0.57 (−0.2, 0.1) ^a^
RIN	6.1 (4.8, 7.3)	5.6 (4.3, 7.0)	0.06 (−5.2 × 10^−5^, 0.9) ^a^	6.2 (4.6, 7.3)	5.5 (2.8, 7.2)	0.03 (0.1, 1.4) ^a^
Uniquely mapped reads in millions	29.7 (25.3, 35.2)	31.0 (15.9, 46.6)	0.64 (−4.1, 2.3) ^a^	29.0 (19.8, 41.1)	28.7 (16.8, 42.2)	0.93 (−4.2, 4.3) ^a^
Uniquely mapped reads %	78.2 (69.0, 83.6)	78.4 (70.6, 86.2)	0.84 (−2.6, 2.6) ^a^	81.7 (78.8, 86.5)	77.2 (62.1, 84.5)	0.01 (0.6, 6.8) ^a^

^a^ *p*-value for Mann–Whitney test with confidence interval for difference in medians. ^b^ *p*-value for Fisher exact test.

**Table 2:** The number of differentially expressed transcripts for cartilage vs. synovium comparisons. The number of differentially expressed transcripts for cartilage vs. synovium comparisons within different treatment groups with different baselines subtracted out. Column labels indicate the treatment group being evaluated, and row labels indicate the baseline being adjusted for. Controls are indicated by “CON”, and pooled surgical groups are indicated by “POOLED”.

	ACLT	RECON	REPAIR	POOLED
CON	329	1210	330	1227
ACLT		97	64	
RECON			99	

**Table 3:** Upregulated biological process GO terms. Biological process GO terms that are upregulated in articular cartilage relative to synovium for each treatment group, adjusting for baseline control differences in tissue type. GO terms are ordered by adjusted *p*-value.

	Upregulated Biological Processes in Cartilage Relative to Synovium	Contributing Genes	Adj. *p*-Value
ACLT	defense response	*CD40LG/PTGFR/FCER1G/CTSC/C5AR1/CCR5/IL2RA/MS4A2/CHI3L1/NKG7/MYD88/GP91-PHOX/CFD/TRIM14/AIF1/IRF5/PIK3CG/TNFAIP8L2*	0.007
	inflammatory response	*CD40LG/PTGFR/CTSC/C5AR1/CCR5/IL2RA/MS4A2/CHI3L1/MYD88/GP91-PHOX/PIK3CG/TNFAIP8L2*	0.007
	chemotaxis	*GDF7/FCER1G/C5AR1/CCR5/LMX1A/PTAFR/AIF1/PIK3CG/VEGFD*	0.046
	taxis	*GDF7/FCER1G/C5AR1/CCR5/LMX1A/PTAFR/AIF1/PIK3CG/VEGFD*	0.046
RECON	system process	*MYMK/CTSC/HTR7/PTPRZ1/AQP3/PTGES/C5AR1/GNAT1/NPY1R/NOS3/P2RY2/TBX20/RGS2/LOC100738836/LUM/HTR1B/NCSTN/LMX1A/CCL2/CNTN5/RAMP2/SMTNL2/SHOX2/F11R/EDNRA/ADRA2A/MYOM1/DRAM2/ADRB2/SCARB1/LHFPL5*	0.023
	vascular process in circulatory system	*HTR7/NOS3/P2RY2/RGS2/HTR1B/RAMP2/SMTNL2/EDNRA/ADRA2A/ADRB2*	0.023
	immune response	*IL7/TNF/CD40LG/CTSC/TNFSF9/FCER1G/BPI/MYD88/TRIM14/CCR5/C5AR1/PTK2B/CYBA/MS4A2/TNFSF15/CCL3L1/C1S/CCL2/IFNAR1/CTSH/GP91-PHOX/SLA-DQB1/SCAP/SCIMP/SLA-DMB/CFD/CCL14/SAMHD1/CD14/POLR3D/MX1/C2/CD74*	0.023
	regulation of tube size	*HTR7/NOS3/P2RY2/RGS2/HTR1B/SMTNL2/EDNRA/ADRA2A/ADRB2*	0.023
	regulation of tube diameter	*HTR7/NOS3/P2RY2/RGS2/HTR1B/SMTNL2/EDNRA/ADRA2A/ADRB2*	0.023
	blood vessel diameter maintenance	*HTR7/NOS3/P2RY2/RGS2/HTR1B/SMTNL2/EDNRA/ADRA2A/ADRB2*	0.023
	defense response	*CD40LG/CHI3L1/CTSC/PTGFR/FCER1G/BPI/MYD88/TNFRSF1A/TRIM14/PIK3CG/CCR5/PTGES/C5AR1/PTK2B/CYBA/IL2RA/MS4A2/NR1H3/C1S/CCL2/IFNAR1/GP91-PHOX/IRF5/APOD/CFD/SAMHD1/CD14/POLR3D/LAPTM5/MX1/C2/CD74*	0.023
	positive regulation of molecular function	*TNF/CD40LG/CHI3L1/TRIM14/PTK2B/SLC5A3/EGF/ARRDC4/NOS3/NLRP3/TNFSF15/ARHGAP45/ADAP2/NCSTN/CCL2/VEGFA/CTSH/EBF2/FAM162A/LAPTM5/RGS1/CAMK2A/ADRA2A/ADRB2/SCARB1/ACVR1C*	0.023
	positive regulation of angiogenesis	*CHI3L1/VEGFD/NRP1/C5AR1/PTK2B/VEGFA/CTSH/RAMP2*	0.023
	positive regulation of vasculature development	*CHI3L1/VEGFD/NRP1/C5AR1/PTK2B/VEGFA/CTSH/RAMP2*	0.023
	cellular response to vascular endothelial growth factor stimulus	*VEGFD/NRP1/DLL4/VEGFA/RAMP2*	0.023
	response to biotic stimulus	*PTGFR/VEGFD/FCER1G/BPI/MYD88/TRIM14/C5AR1/PTK2B/CYBA/NOS3/C1S/CCL2/IFNAR1/GP91-PHOX/IRF5/THRSP/SCIMP/SLC11A1/CFD/SAMHD1/CD14/POLR3D/LAPTM5/RGS1/MX1/C2/SCARB1/SRPX*	0.023
	receptor-mediated endocytosis	*FCER1G/APLN/MSR1/VEGFA/RAMP2/CBL/MRC1/ITGB2/ADRB2*	0.032
	response to bacterium	*PTGFR/VEGFD/FCER1G/BPI/MYD88/C5AR1/NOS3/CCL2/IFNAR1/THRSP/SCIMP/SLC11A1/CFD/RGS1/SCARB1*	0.032
	sprouting angiogenesis	*VEGFD/NRP1/DLL4/PTK2B/VEGFA/RAMP2/ESM1*	0.034
	blood circulation	*HTR7/NPY1R/NOS3/P2RY2/TBX20/RGS2/HTR1B/RAMP2/SMTNL2/SHOX2/EDNRA/ADRA2A/ADRB2*	0.040
	positive regulation of signal transduction	*TNF/CHI3L1/CTSC/HHEX/IL10RA/MYD88/NRP1/S100A4/DLL4/C5AR1/PTK2B/TBX20/RASGRP4/LOC100738836/CCL2/VEGFA/CTSH/SHOX2/SCIMP/CBL/NR3C2/ESM1/ADRA2A/ADRB2/CD74/SRPX*	0.042
	positive regulation of catalytic activity	*CHI3L1/PTK2B/SLC5A3/ARRDC4/NOS3/NLRP3/TNFSF15/ARHGAP45/ADAP2/NCSTN/CCL2/VEGFA/CTSH/FAM162A/LAPTM5/RGS1/ADRA2A/ADRB2/SCARB1/ACVR1C*	0.042
	regulation of cell migration	*VEGFD/PHACTR1/NRP1/RAP2B/DLL4/C5AR1/PTK2B/ARHGDIB/LOC100738836/CCL2/VEGFA/CTSH/APOD/CAMK2A/ADRA2A/TMSB4X/ACVR1C*	0.042
	response to other organism	*PTGFR/VEGFD/FCER1G/BPI/MYD88/TRIM14/C5AR1/PTK2B/CYBA/NOS3/C1S/CCL2/IFNAR1/GP91-PHOX/IRF5/THRSP/SCIMP/SLC11A1/CFD/SAMHD1/CD14/POLR3D/RGS1/MX1/C2/SCARB1*	0.042
	vascular endothelial growth factor receptor signaling pathway	*VEGFD/NRP1/PTK2B/VEGFA*	0.042
	response to external biotic stimulus	*PTGFR/VEGFD/FCER1G/BPI/MYD88/TRIM14/C5AR1/PTK2B/CYBA/NOS3/C1S/CCL2/IFNAR1/GP91-PHOX/IRF5/THRSP/SCIMP/SLC11A1/CFD/SAMHD1/CD14/POLR3D/RGS1/MX1/C2/SCARB1*	0.042
	actin cytoskeleton reorganization	*PHACTR1/NRP1/RAP2B/ESAM/PTK2B/ARHGDIB*	0.043
	inflammatory response	*CD40LG/CHI3L1/CTSC/PTGFR/MYD88/TNFRSF1A/PIK3CG/CCR5/PTGES/C5AR1/IL2RA/MS4A2/NR1H3/CCL2/GP91-PHOX/APOD/CD14/CD74*	0.044
	circulatory system process	*HTR7/NPY1R/NOS3/P2RY2/TBX20/RGS2/HTR1B/RAMP2/SMTNL2/SHOX2/EDNRA/ADRA2A/ADRB2*	0.044
	biological process involved in interspecies interaction between organisms	*PTGFR/VEGFD/FCER1G/BPI/MYD88/NRP1/TRIM14/C5AR1/PTK2B/CYBA/NOS3/C1S/CCL2/IFNAR1/GP91-PHOX/IRF5/THRSP/SCIMP/SLC11A1/CFD/SAMHD1/CD14/POLR3D/RGS1/MX1/C2/SCARB1*	0.044
	positive regulation of cell communication	*TNF/CHI3L1/CTSC/HHEX/IL10RA/MYD88/NRP1/S100A4/DLL4/C5AR1/PTK2B/TBX20/RASGRP4/LOC100738836/NCSTN/CCL2/VEGFA/CTSH/SHOX2/SCIMP/CBL/NR3C2/ESM1/ADRA2A/ADRB2/CD74/SRPX*	0.049
	positive regulation of peptidase activity	*NLRP3/TNFSF15/NCSTN/CTSH/FAM162A/LAPTM5/ACVR1C*	0.049
REPAIR			

**Table 4:** Upregulated molecular function GO terms. Molecular function GO terms that are upregulated in articular cartilage relative to synovium for each treatment group, adjusting for baseline control differences in tissue type. GO terms are ordered by adjusted *p*-value.

	Upregulated Molecular Functions in Cartilage Relative to Synovium	Contributing Genes	Adj. *p*-Value
ACLT			
RECON	signaling receptor activity	*NPY5R/PTGFR/EFEMP1/FCER1G/HTR7/IL10RA/TNFRSF1A/PRTG/NRP1/PECAM1/CCR5/C5AR1/NPY1R/IL2RA/P2RY2/NR1H3/NR5A2/LOC100737531/PTGDR2/HTR1B/IFNAR1/CTSH/GPR34/NOTCH4/RAMP2/FZD4/P2RY6/RORC/MRC1/P2RY12/ITGB2/CNTFR/EDNRA/ADRA2A/ADRB2/SCARB1/ACVR1C/PTAFR*	0.001
	molecular transducer activity	*NPY5R/PTGFR/EFEMP1/FCER1G/HTR7/IL10RA/TNFRSF1A/PRTG/NRP1/PECAM1/CCR5/C5AR1/NPY1R/IL2RA/P2RY2/NR1H3/NR5A2/LOC100737531/PTGDR2/HTR1B/IFNAR1/CTSH/GPR34/NOTCH4/RAMP2/FZD4/P2RY6/RORC/MRC1/P2RY12/ITGB2/CNTFR/EDNRA/ADRA2A/ADRB2/SCARB1/ACVR1C/PTAFR*	0.001
	enzyme activator activity	*CD40LG/CTSC/PCOLCE/NRP1/GIT2/IGFBP3/SMAP2/NCF2/RGS2/ARHGAP45/ADAP2/CTSH/ARHGAP25/RGS1/NCF4/FGL2*	0.001
	transmembrane signaling receptor activity	*NPY5R/PTGFR/EFEMP1/FCER1G/HTR7/IL10RA/TNFRSF1A/NRP1/PECAM1/CCR5/C5AR1/NPY1R/IL2RA/P2RY2/LOC100737531/PTGDR2/HTR1B/IFNAR1/CTSH/GPR34/RAMP2/FZD4/P2RY6/P2RY12/CNTFR/EDNRA/ADRA2A/ADRB2/ACVR1C/PTAFR*	0.010
	GTPase activator activity	*NRP1/GIT2/SMAP2/RGS2/ARHGAP45/ADAP2/ARHGAP25/RGS1*	0.032
	purinergic nucleotide receptor activity	*P2RY2/GPR34/P2RY6/P2RY12*	0.032
	nucleotide receptor activity	*P2RY2/GPR34/P2RY6/P2RY12*	0.032
	immune receptor activity	*FCER1G/IL10RA/CCR5/C5AR1/IL2RA/IFNAR1/CTSH/CNTFR*	0.034
	carbohydrate transmembrane transporter activity	*AQP3/SLC5A3/TMEM144/AQP9*	0.046
	peptidase activator activity	*CTSC/PCOLCE/CTSH/FGL2*	0.046
REPAIR	transmembrane signaling receptor activity	*HTR7/EFEMP1/GABRE/FCER1G/PTGFR/P2RY6/C5AR1/PTAFR/CCR5/IL2RA/CD300C*	0.034
	signaling receptor activity	*PRTG/HTR7/EFEMP1/GABRE/FCER1G/PTGFR/P2RY6/C5AR1/PTAFR/CCR5/IL2RA/CD300C*	0.034
	molecular transducer activity	*PRTG/HTR7/EFEMP1/GABRE/FCER1G/PTGFR/P2RY6/C5AR1/PTAFR/CCR5/IL2RA/CD300C*	0.034
	cytokine receptor binding	*IL7/TNF/CD40LG/CCL3L1/VEGFA/VEGFD*	0.034
	immune receptor activity	*FCER1G/C5AR1/CCR5/IL2RA*	0.034
	cytokine activity	*IL7/GDF7/TNF/CD40LG/CCL3L1/VEGFA*	0.034
	receptor ligand activity	*IL7/GDF7/TNF/CD40LG/CCL3L1/VEGFA/APLN/VEGFD*	0.034
	signaling receptor activator activity	*IL7/GDF7/TNF/CD40LG/CCL3L1/VEGFA/APLN/VEGFD*	0.034
	signaling receptor regulator activity	*IL7/GDF7/TNF/CD40LG/CCL3L1/VEGFA/APLN/VEGFD*	0.037

## Data Availability

The data presented in this study are openly available through the appendices mentioned in the main text. RNA sequencing fastq files, Salmon files, and metadata used for analysis are available through NCBI’s GEO database with the accession number GSE228848.

## Supplementary Materials

The following supporting information can be downloaded at https://www.mdpi.com/article/10.3390/bioengineering10050527/s1. Supplement S1: Detailed Animal Procedures. Supplement S2: MultiQC Reports. Supplement S3: Demographics and RNA statistics. Supplement S4: PCA Outliers. Refs. [56,57,58,59,60] can be found in the supplementary materials.
