# Supplementary material for: Responding to ACL Injury and its Treatments: Comparative Gene Expression between Articular Cartilage and Synovium
Source: Bioengineering (Basel). 2023 Apr 26;10(5):527. doi: 10.3390/bioengineering10050527 (PMC10215325; doi:10.3390/bioengineering10050527)

MultiQC

(http://multiqc.info)

A modular tool to aggregate results from bioinformatics analyses across many samples into a single report.

Report generated on 2022-08-15, 21:53 based on data in:

- /n/scratch3/users/j/jid4/Articular_Cartilage_long_term/fastqc_results/LIB053209_TRA00222266_S49_R1_001_fastqc.html
- /n/scratch3/users/j/jid4/Articular_Cartilage_long_term/fastqc_results/LIB053209_TRA00222266_S49_R1_001_fastqc.zip
- [illegible]

General Statistics

Articular Cartilage

Copy table | Configure Columns | Plot | Showing 96/96 rows and 3/5 columns.

Sample Name	% Dups	% GC	M Seqs
LIB053209_TRA00222266_S49_R1_001			
LIB053209_TRA00222266_S49_R2_001			
LIB053209_TRA00222267_S50_R1_001			
LIB053209_TRA00222267_S50_R2_001			
LIB053209_TRA00222268_S51_R1_001			
LIB053209_TRA00222268_S51_R2_001			
LIB053209_TRA00222269_S52_R1_001			
LIB053209_TRA00222269_S52_R2_001			
LIB053209_TRA00222270_S53_R1_001			
LIB053209_TRA00222270_S53_R2_001			
LIB053209_TRA00222271_S54_R1_001			
LIB053209_TRA00222271_S54_R2_001			
LIB053209_TRA00222272_S55_R1_001			
LIB053209_TRA00222272_S55_R2_001			
LIB053209_TRA00222273_S56_R1_001			
LIB053209_TRA00222273_S56_R2_001			
LIB053209_TRA00222274_S57_R1_001			
LIB053209_TRA00222274_S57_R2_001			
LIB053209_TRA00222275_S58_R1_001			
LIB053209_TRA00222275_S58_R2_001			
LIB053209_TRA00222276_S59_R1_001			
LIB053209_TRA00222276_S59_R2_001			
LIB053209_TRA00222277_S60_R1_001			
LIB053209_TRA00222277_S60_R2_001			
LIB053209_TRA00222278_S61_R1_001			
LIB053209_TRA00222278_S61_R2_001			

Sample Name	% Dups	% GC	M Seqs
LIB053209_TRA00222279_S62_R1_001			
LIB053209_TRA00222279_S62_R2_001			
LIB053209_TRA00222280_S63_R1_001			
LIB053209_TRA00222280_S63_R2_001			
LIB053209_TRA00222281_S64_R1_001			
LIB053209_TRA00222281_S64_R2_001			
LIB053209_TRA00222282_S65_R1_001			
LIB053209_TRA00222282_S65_R2_001			
LIB053209_TRA00222283_S66_R1_001			
LIB053209_TRA00222283_S66_R2_001			
LIB053209_TRA00222284_S67_R1_001			
LIB053209_TRA00222284_S67_R2_001			
LIB053209_TRA00222285_S68_R1_001			
LIB053209_TRA00222285_S68_R2_001			
LIB053209_TRA00222286_S69_R1_001			
LIB053209_TRA00222286_S69_R2_001			
LIB053209_TRA00222287_S70_R1_001			
LIB053209_TRA00222287_S70_R2_001			
LIB053209_TRA00222288_S71_R1_001			
LIB053209_TRA00222288_S71_R2_001			
LIB053209_TRA00222289_S72_R1_001			
LIB053209_TRA00222289_S72_R2_001			
LIB053209_TRA00222290_S73_R1_001			
LIB053209_TRA00222290_S73_R2_001			
LIB053209_TRA00222291_S74_R1_001			
LIB053209_TRA00222291_S74_R2_001			
LIB053209_TRA00222292_S75_R1_001			
LIB053209_TRA00222292_S75_R2_001			
LIB053209_TRA00222293_S76_R1_001			
LIB053209_TRA00222293_S76_R2_001			
LIB053209_TRA00222294_S77_R1_001			
LIB053209_TRA00222294_S77_R2_001			
LIB053209_TRA00222295_S78_R1_001			
LIB053209_TRA00222295_S78_R2_001			
LIB053209_TRA00222296_S79_R1_001			
LIB053209_TRA00222296_S79_R2_001			
LIB053209_TRA00222297_S80_R1_001			
LIB053209_TRA00222297_S80_R2_001			
LIB053209_TRA00222298_S81_R1_001			
LIB053209_TRA00222298_S81_R2_001			
LIB053209_TRA00222299_S82_R1_001			
LIB053209_TRA00222299_S82_R2_001			
LIB053209_TRA00222300_S83_R1_001			
LIB053209_TRA00222300_S83_R2_001			
LIB053209_TRA00222301_S84_R1_001			
LIB053209_TRA00222301_S84_R2_001			

Sample Name	% Dups	% GC	M Seqs
LIB053209_TRA00222302_S85_R1_001			
LIB053209_TRA00222302_S85_R2_001			
LIB053209_TRA00222303_S86_R1_001			
LIB053209_TRA00222303_S86_R2_001			
LIB053209_TRA00222304_S87_R1_001			
LIB053209_TRA00222304_S87_R2_001			
LIB053209_TRA00222305_S88_R1_001			
LIB053209_TRA00222305_S88_R2_001			
LIB053209_TRA00222306_S89_R1_001			
LIB053209_TRA00222306_S89_R2_001			
LIB053209_TRA00222307_S90_R1_001			
LIB053209_TRA00222307_S90_R2_001			
LIB053209_TRA00222308_S91_R1_001			
LIB053209_TRA00222308_S91_R2_001			
LIB053209_TRA00222309_S92_R1_001			
LIB053209_TRA00222309_S92_R2_001			
LIB053209_TRA00222310_S93_R1_001			
LIB053209_TRA00222310_S93_R2_001			
LIB053209_TRA00222311_S94_R1_001			
LIB053209_TRA00222311_S94_R2_001			
LIB053209_TRA00222312_S95_R1_001			
LIB053209_TRA00222312_S95_R2_001			
LIB053209_TRA00222313_S96_R1_001			
LIB053209_TRA00222313_S96_R2_001			

FastQC

FastQC (http://www.bioinformatics.babraham.ac.uk/projects/fastqc/) is a quality control tool for high throughput sequence data, written by Simon Andrews at the Babraham Institute in Cambridge.

Sequence Counts

Help

Sequence counts for each sample. Duplicate read counts are an estimate only.

Number of reads | Percentages

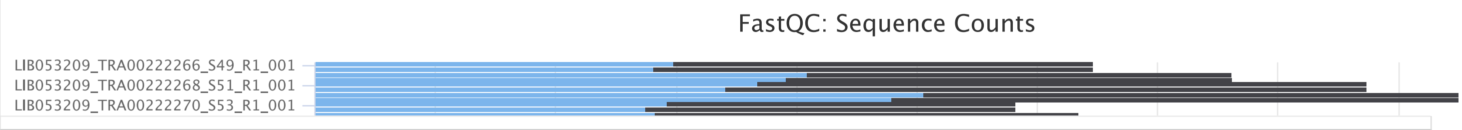

Sequence Quality Histograms 96

Help

The mean quality value across each base position in the read.

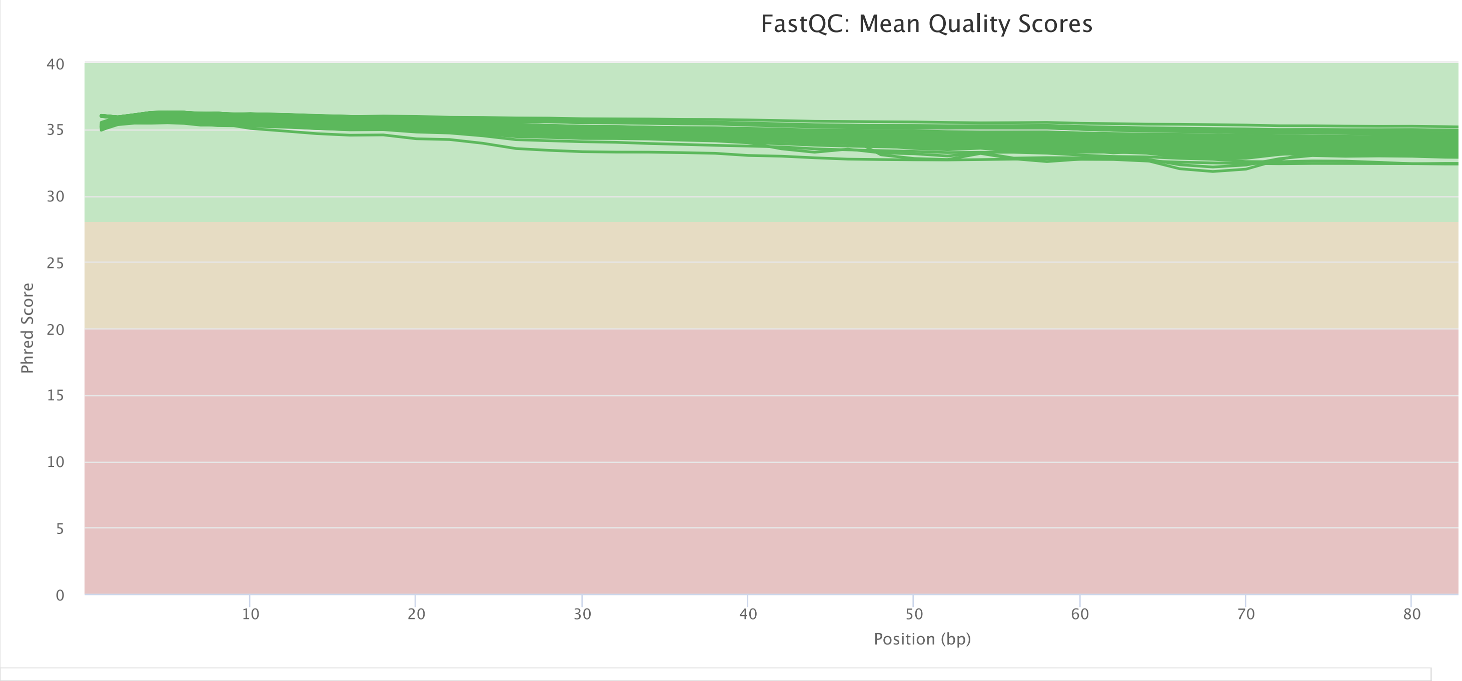

Per Sequence Quality Scores 96

Help

The number of reads with average quality scores. Shows if a subset of reads has poor quality.

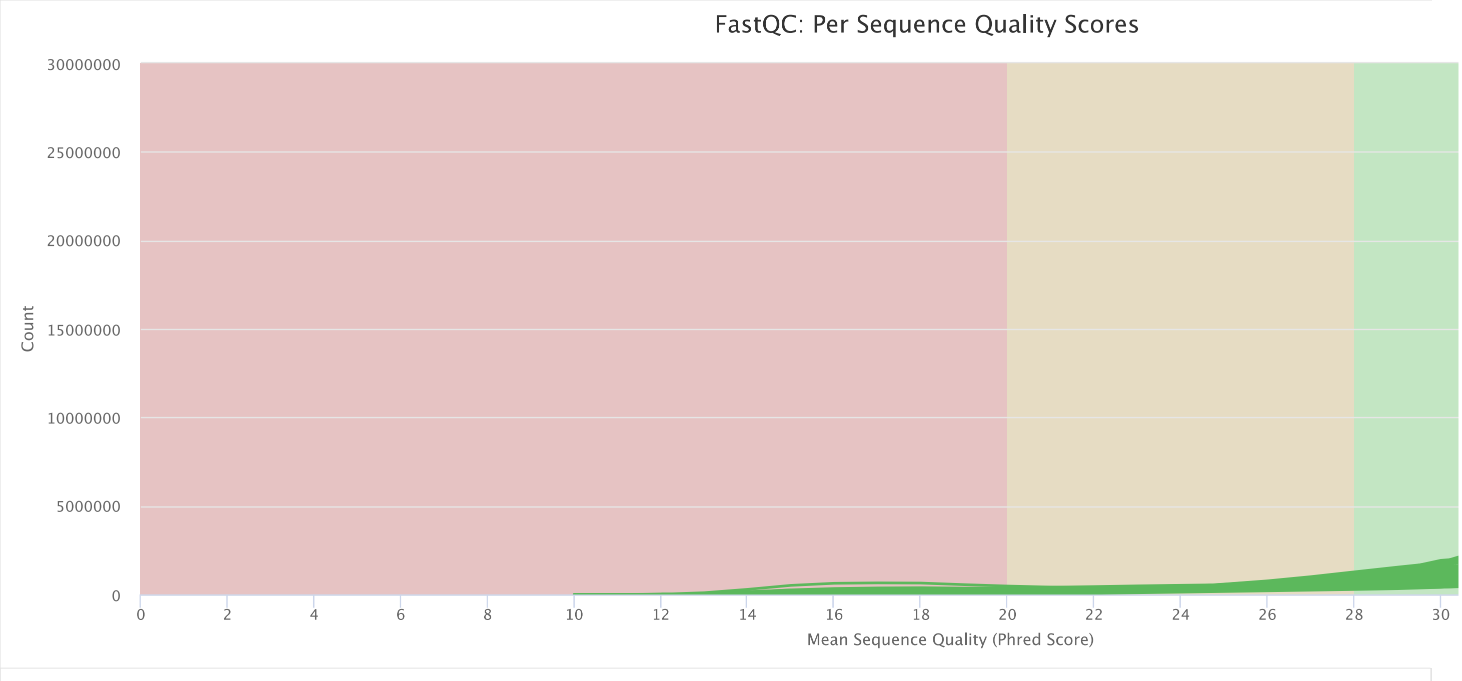

Per Base Sequence Content 0 96

Help

The proportion of each base position for which each of the four normal DNA bases has been called.

Click a sample row to see a line plot for that dataset.

Rollover for sample name

Position: - %T: - %C: - %A: - %G: - Export Plot

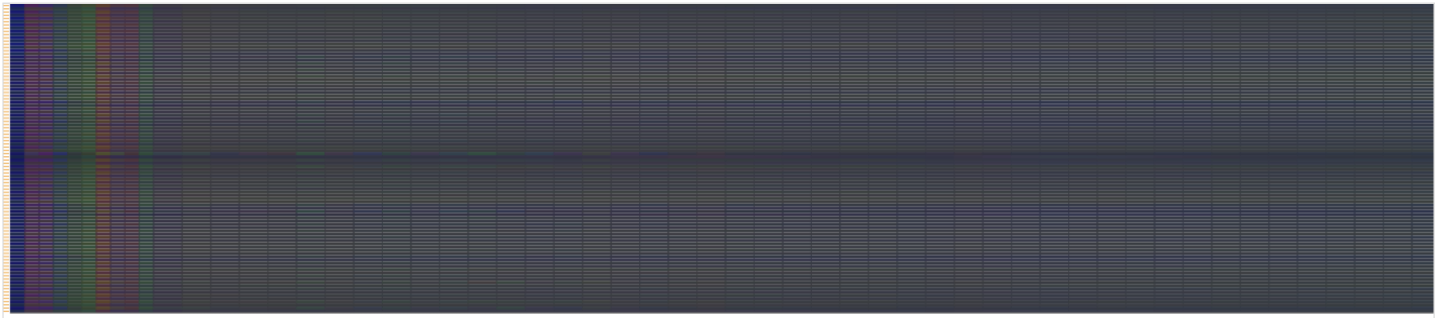

Per Sequence GC Content 20 76

Help

The average GC content of reads. Normal random library typically have a roughly normal distribution of GC content.

Percentages | Counts

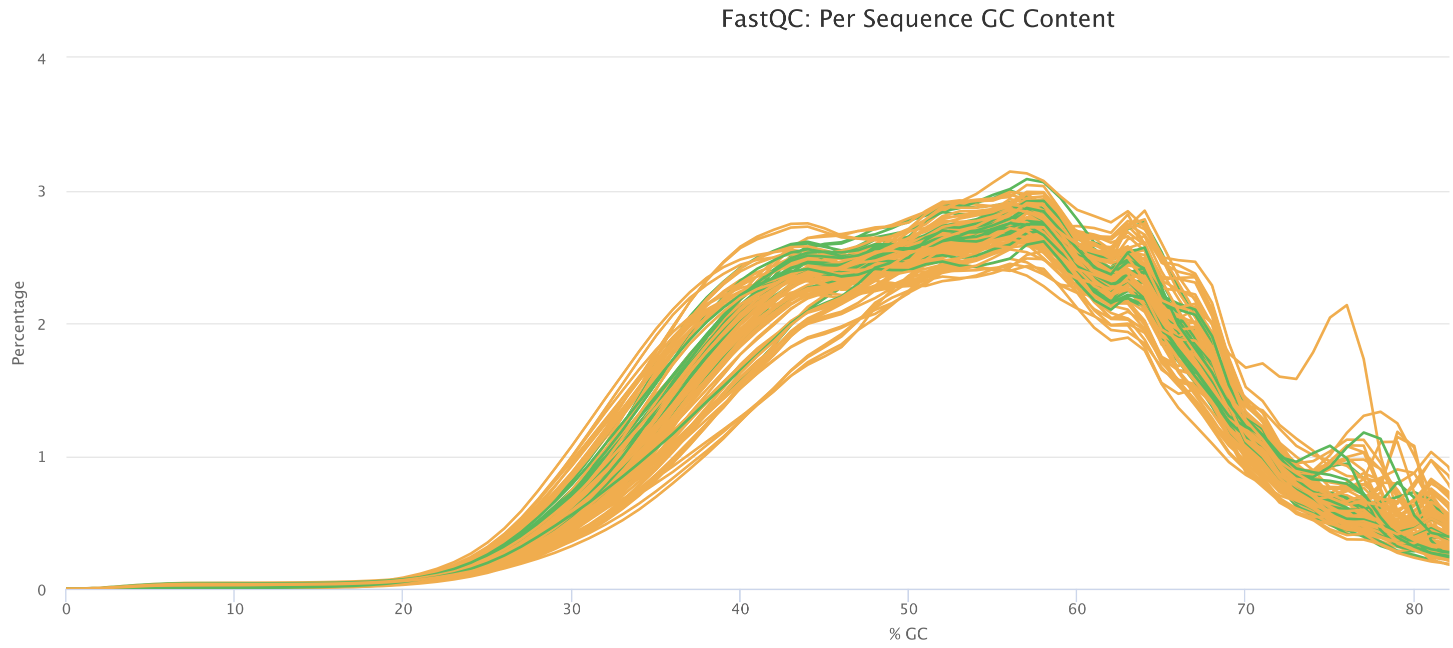

Per Base N Content 96

Help

The percentage of base calls at each position for which an `N` was called.

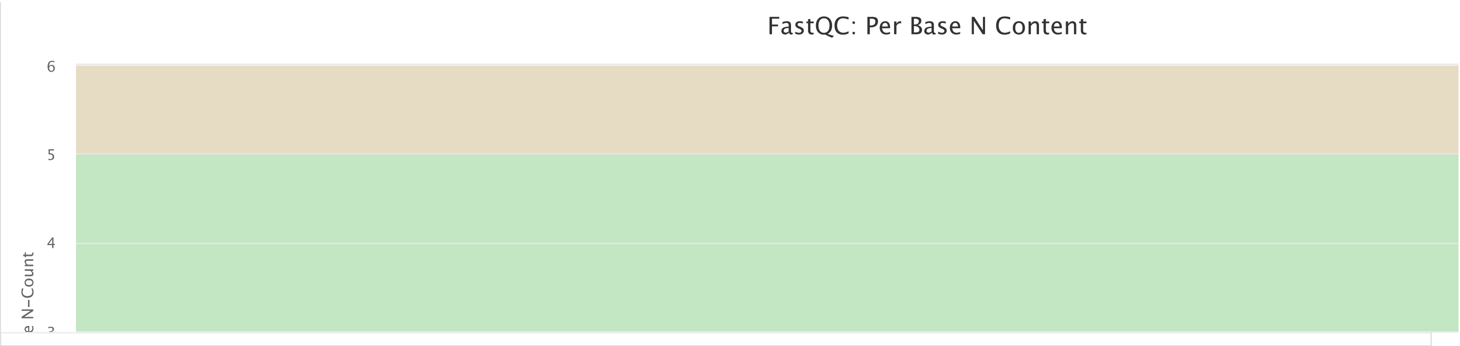

Sequence Length Distribution 96

All samples have sequences of a single length (100bp).

Sequence Duplication Levels 0 68 28

Help

The relative level of duplication found for every sequence.

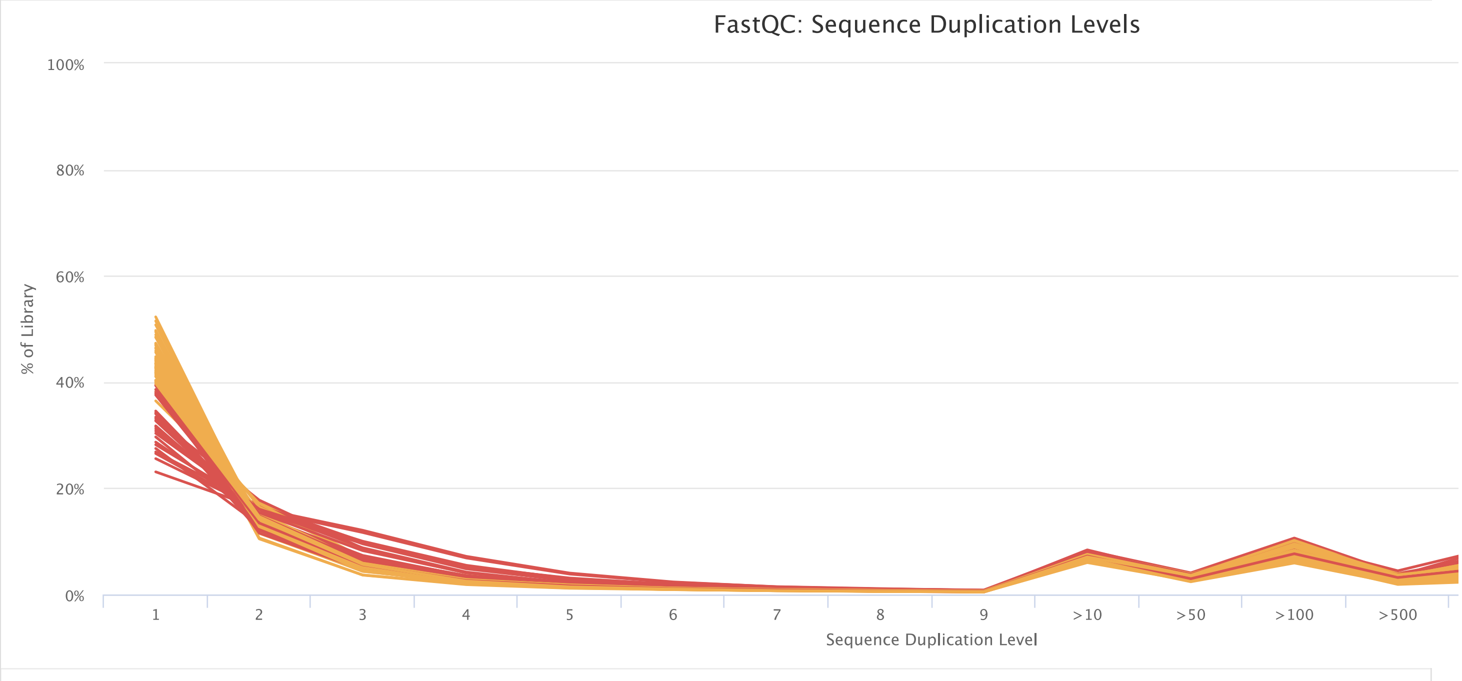

Overrepresented sequences 0 86 1(

Help

The total amount of overrepresented sequences found in each library.

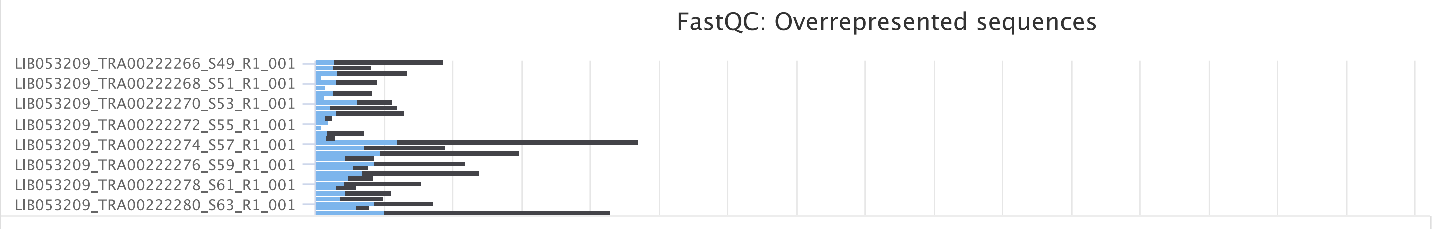

Adapter Content 20 76

Help

The cumulative percentage count of the proportion of your library which has seen each of the adapter sequences at each position.

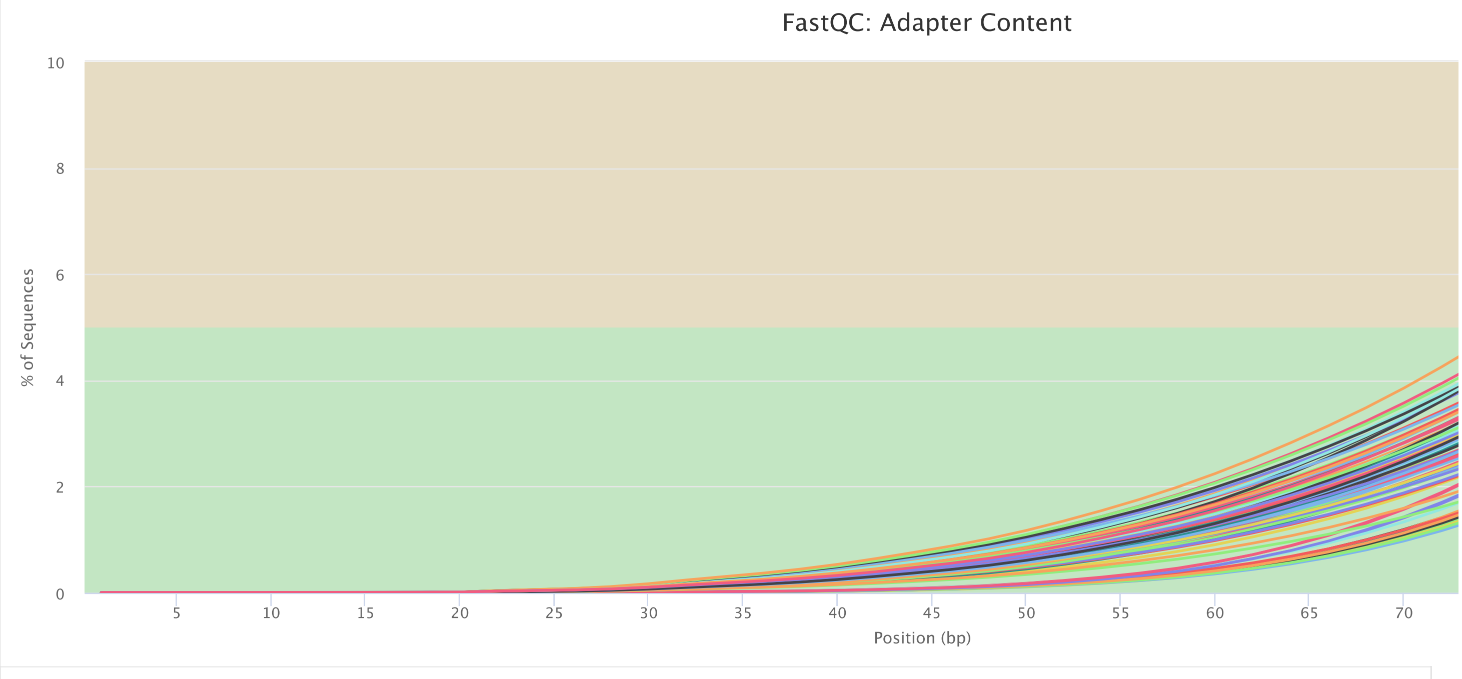

Status Checks

Help

Status for each FastQC section showing whether results seem entirely normal (green), slightly abnormal (orange) or very unusual (red).

Sort by highlight

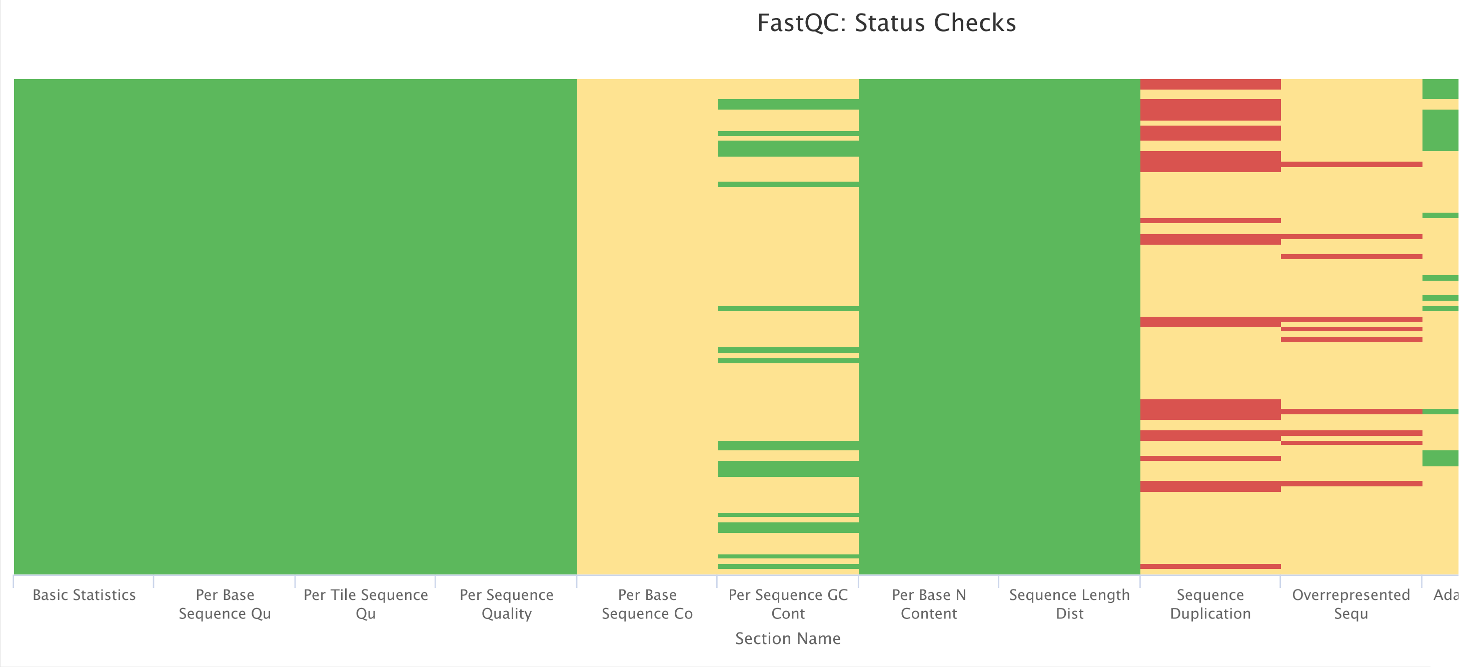

MultiQC v1.9 (http://multiqc.info) - Written by Phil Ewels (http://phil.ewels.co.uk), available on GitHub (https://github.com/ewels/MultiQC).
This report uses HighCharts (http://www.highcharts.com/), jQuery (https://jquery.com/), jQuery UI (https://jqueryui.com/), Bootstrap (http://getbootstrap.com/), FileSaver.js (https://github.com/eligrey/FileSaver.js) and clipboard.js (https://clipboardjs.com/).

SciLifeLab (http://www.scilifelab.se/)

(http://multiqc.info)

A modular tool to aggregate results from bioinformatics analyses across many samples into a single report.

Synovium

Report generated on 2022-08-15, 21:57 based on data in:

- /n/scratch3/users/j/jid4/Synovium_long_term/fastqc_results/LIB053209_TRA00222218_S1_R1_001_fastqc.html
- /n/scratch3/users/j/jid4/Synovium_long_term/fastqc_results/LIB053209_TRA00222218_S1_R1_001_fastqc.zip
- [illegible]

General Statistics

Copy table | Configure Columns | Plot | Showing 96/96 rows and 3/5 columns.

Sample Name	% Dups	% GC	M Seqs
LIB053209_TRA00222218_S1_R1_001			
LIB053209_TRA00222218_S1_R2_001			
LIB053209_TRA00222219_S2_R1_001			
LIB053209_TRA00222219_S2_R2_001			
LIB053209_TRA00222220_S3_R1_001			
LIB053209_TRA00222220_S3_R2_001			
LIB053209_TRA00222221_S4_R1_001			
LIB053209_TRA00222221_S4_R2_001			
LIB053209_TRA00222222_S5_R1_001			
LIB053209_TRA00222222_S5_R2_001			
LIB053209_TRA00222223_S6_R1_001			
LIB053209_TRA00222223_S6_R2_001			
LIB053209_TRA00222224_S7_R1_001			
LIB053209_TRA00222224_S7_R2_001			
LIB053209_TRA00222225_S8_R1_001			
LIB053209_TRA00222225_S8_R2_001			
LIB053209_TRA00222226_S9_R1_001			
LIB053209_TRA00222226_S9_R2_001			
LIB053209_TRA00222227_S10_R1_001			
LIB053209_TRA00222227_S10_R2_001			
LIB053209_TRA00222228_S11_R1_001			
LIB053209_TRA00222228_S11_R2_001			
LIB053209_TRA00222229_S12_R1_001			
LIB053209_TRA00222229_S12_R2_001			
LIB053209_TRA00222230_S13_R1_001			
LIB053209_TRA00222230_S13_R2_001			

Sample Name	% Dups	% GC	M Seqs
LIB053209_TRA00222231_S14_R1_001			
LIB053209_TRA00222231_S14_R2_001			
LIB053209_TRA00222232_S15_R1_001			
LIB053209_TRA00222232_S15_R2_001			
LIB053209_TRA00222233_S16_R1_001			
LIB053209_TRA00222233_S16_R2_001			
LIB053209_TRA00222234_S17_R1_001			
LIB053209_TRA00222234_S17_R2_001			
LIB053209_TRA00222235_S18_R1_001			
LIB053209_TRA00222235_S18_R2_001			
LIB053209_TRA00222236_S19_R1_001			
LIB053209_TRA00222236_S19_R2_001			
LIB053209_TRA00222237_S20_R1_001			
LIB053209_TRA00222237_S20_R2_001			
LIB053209_TRA00222238_S21_R1_001			
LIB053209_TRA00222238_S21_R2_001			
LIB053209_TRA00222239_S22_R1_001			
LIB053209_TRA00222239_S22_R2_001			
LIB053209_TRA00222240_S23_R1_001			
LIB053209_TRA00222240_S23_R2_001			
LIB053209_TRA00222241_S24_R1_001			
LIB053209_TRA00222241_S24_R2_001			
LIB053209_TRA00222242_S25_R1_001			
LIB053209_TRA00222242_S25_R2_001			
LIB053209_TRA00222243_S26_R1_001			
LIB053209_TRA00222243_S26_R2_001			
LIB053209_TRA00222244_S27_R1_001			
LIB053209_TRA00222244_S27_R2_001			
LIB053209_TRA00222245_S28_R1_001			
LIB053209_TRA00222245_S28_R2_001			
LIB053209_TRA00222246_S29_R1_001			
LIB053209_TRA00222246_S29_R2_001			
LIB053209_TRA00222247_S30_R1_001			
LIB053209_TRA00222247_S30_R2_001			
LIB053209_TRA00222248_S31_R1_001			
LIB053209_TRA00222248_S31_R2_001			
LIB053209_TRA00222249_S32_R1_001			
LIB053209_TRA00222249_S32_R2_001			
LIB053209_TRA00222250_S33_R1_001			
LIB053209_TRA00222250_S33_R2_001			
LIB053209_TRA00222251_S34_R1_001			
LIB053209_TRA00222251_S34_R2_001			
LIB053209_TRA00222252_S35_R1_001			
LIB053209_TRA00222252_S35_R2_001			
LIB053209_TRA00222253_S36_R1_001			
LIB053209_TRA00222253_S36_R2_001			

Sample Name	% Dups	% GC	M Seqs
**LIB053209_TRA00222254_S37_R1_001**			
**LIB053209_TRA00222254_S37_R2_001**			
**LIB053209_TRA00222255_S38_R1_001**			
**LIB053209_TRA00222255_S38_R2_001**			
**LIB053209_TRA00222256_S39_R1_001**			
**LIB053209_TRA00222256_S39_R2_001**			
**LIB053209_TRA00222257_S40_R1_001**			
**LIB053209_TRA00222257_S40_R2_001**			
**LIB053209_TRA00222258_S41_R1_001**			
**LIB053209_TRA00222258_S41_R2_001**			
**LIB053209_TRA00222259_S42_R1_001**			
**LIB053209_TRA00222259_S42_R2_001**			
**LIB053209_TRA00222260_S43_R1_001**			
**LIB053209_TRA00222260_S43_R2_001**			
**LIB053209_TRA00222261_S44_R1_001**			
**LIB053209_TRA00222261_S44_R2_001**			
**LIB053209_TRA00222262_S45_R1_001**			
**LIB053209_TRA00222262_S45_R2_001**			
**LIB053209_TRA00222263_S46_R1_001**			
**LIB053209_TRA00222263_S46_R2_001**			
**LIB053209_TRA00222264_S47_R1_001**			
**LIB053209_TRA00222264_S47_R2_001**			
**LIB053209_TRA00222265_S48_R1_001**			
**LIB053209_TRA00222265_S48_R2_001**			

FastQC

FastQC (http://www.bioinformatics.babraham.ac.uk/projects/fastqc/) is a quality control tool for high throughput sequence data, written by Simon Andrews at the Babraham Institute in Cambridge.

Sequence Counts

Help

Sequence counts for each sample. Duplicate read counts are an estimate only.

Number of reads | Percentages

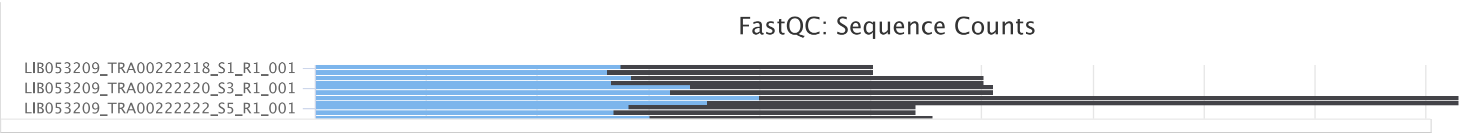

Sequence Quality Histograms 96

Help

The mean quality value across each base position in the read.

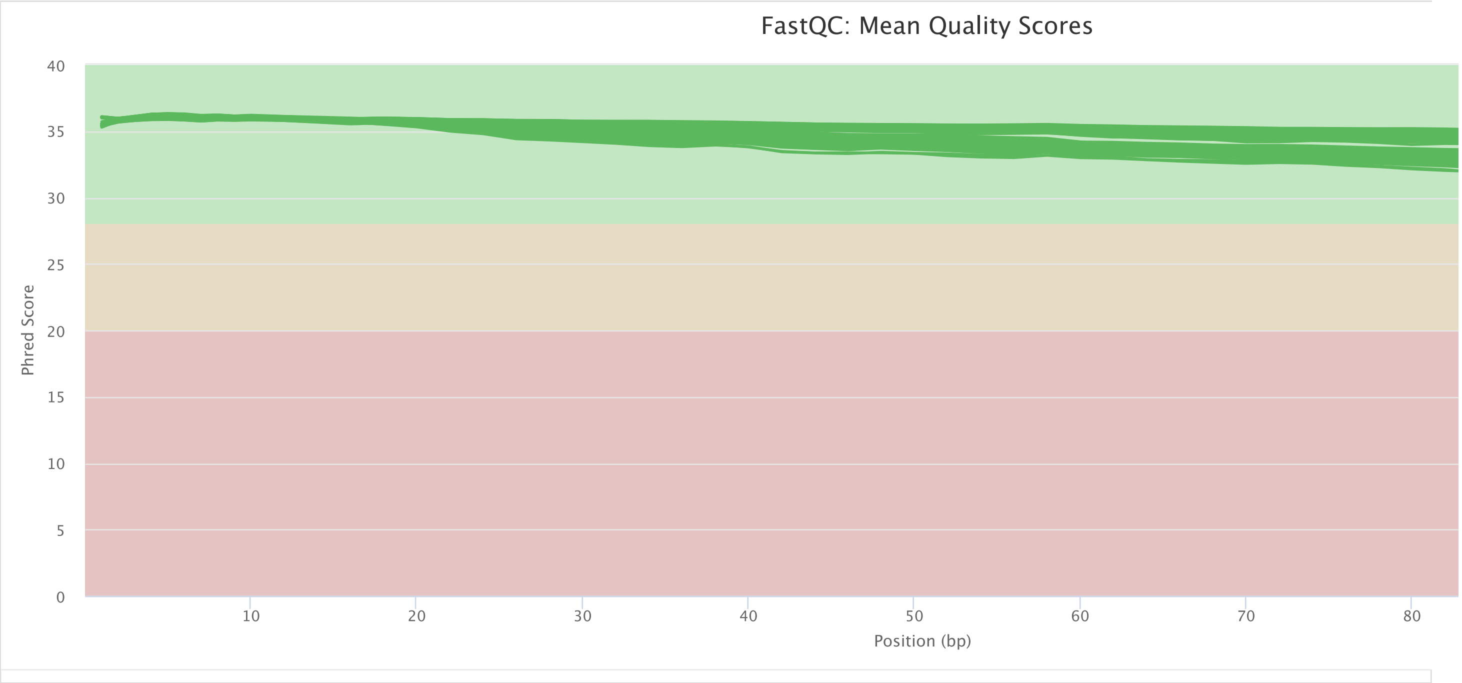

Per Sequence Quality Scores 96

Help

The number of reads with average quality scores. Shows if a subset of reads has poor quality.

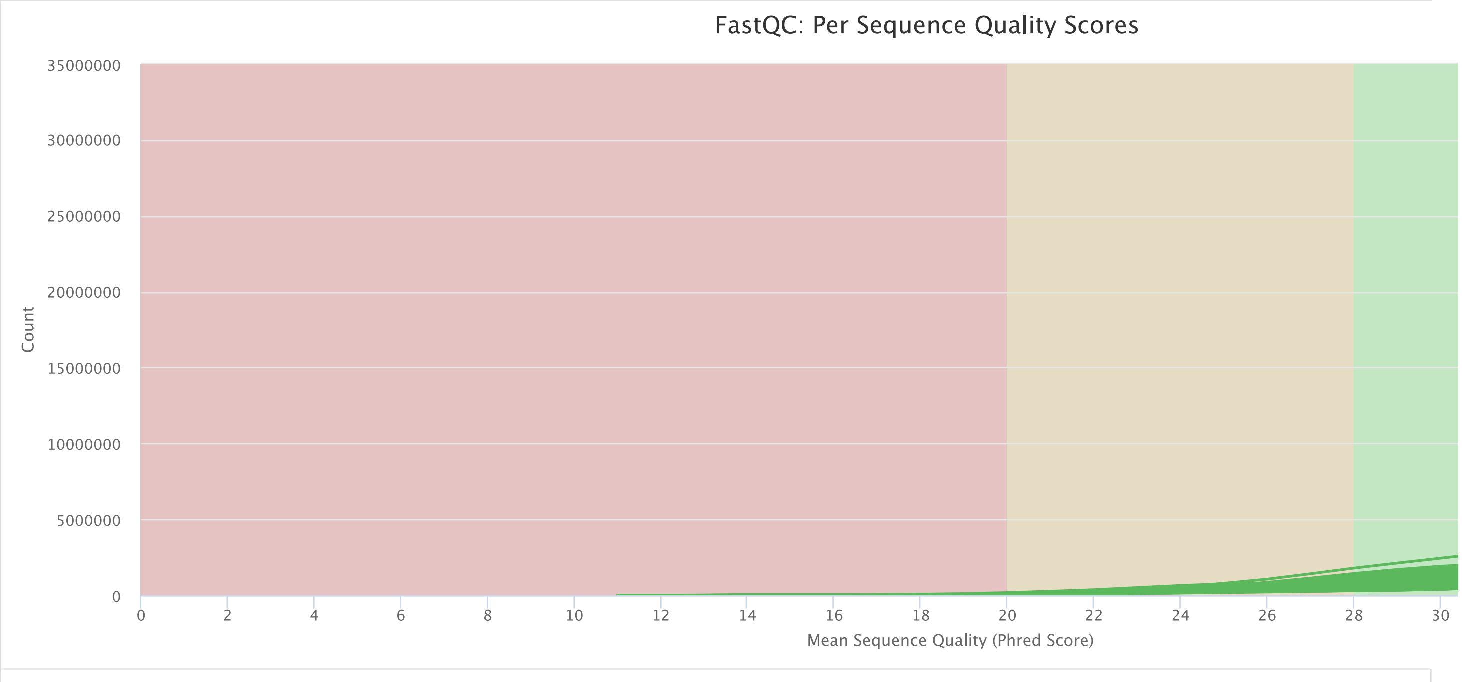

Per Base Sequence Content 0 96

Help

The proportion of each base position for which each of the four normal DNA bases has been called.

Click a sample row to see a line plot for that dataset.

Rollover for sample name

Position: - %T: - %C: - %A: - %G: -

Export Plot

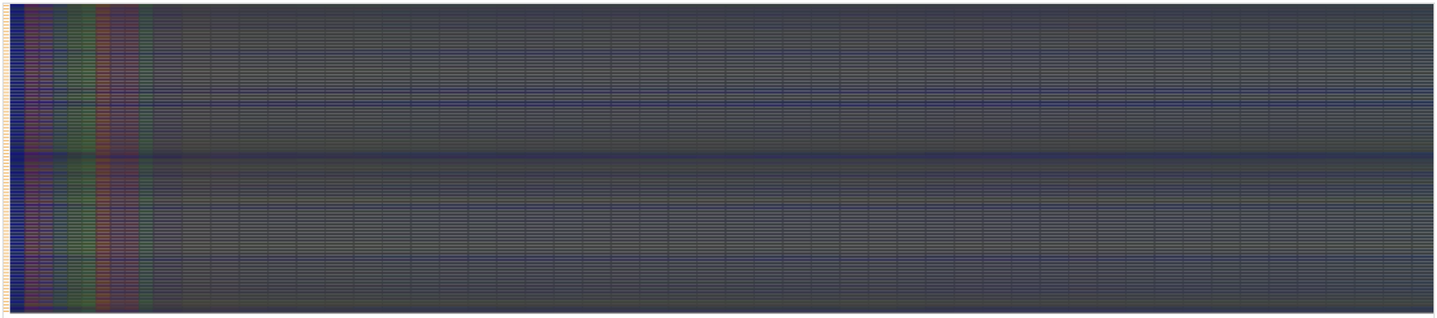

Per Sequence GC Content 3 39 54

Help

The average GC content of reads. Normal random library typically have a roughly normal distribution of GC content.

Percentages Counts

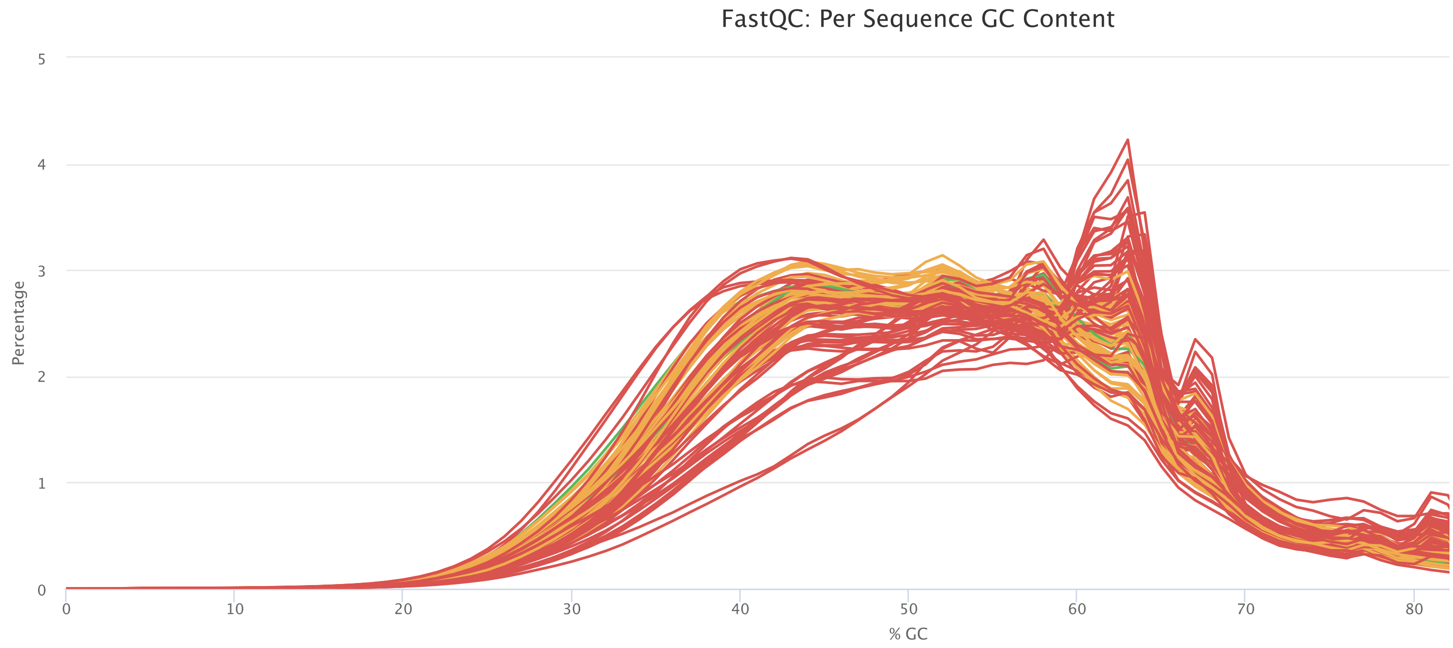

Per Base N Content 96

Help

The percentage of base calls at each position for which an N was called.

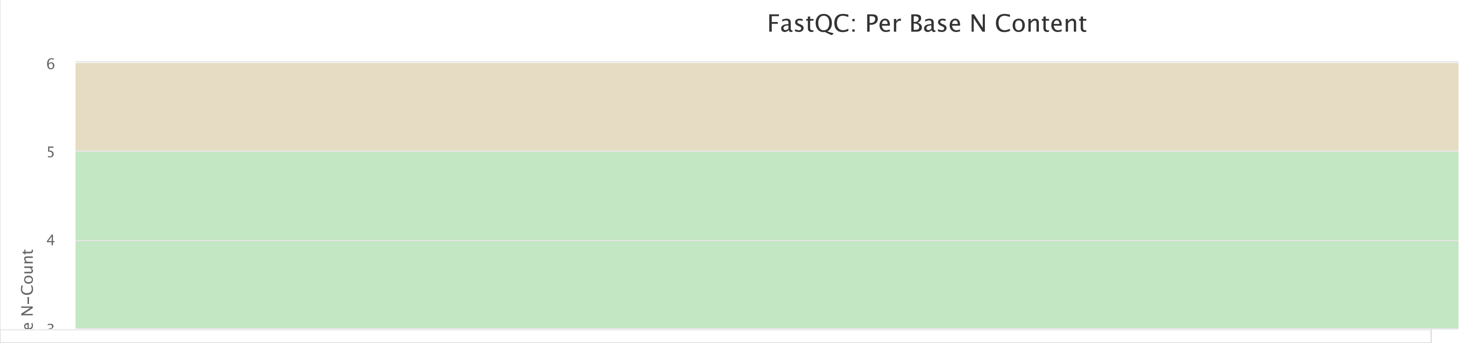

Sequence Length Distribution 96

All samples have sequences of a single length (100bp).

Sequence Duplication Levels 0 40 56

Help

The relative level of duplication found for every sequence.

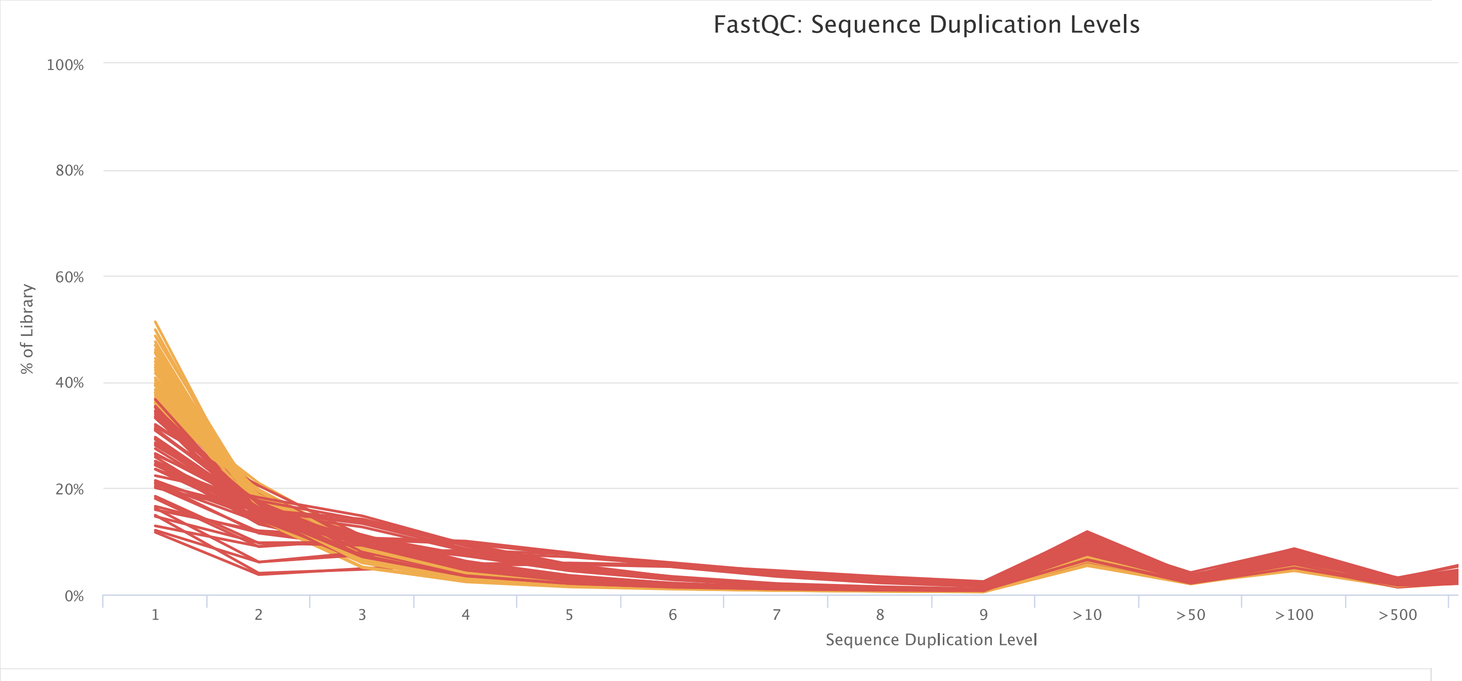

Overrepresented sequences 0 90 6

Help

The total amount of overrepresented sequences found in each library.

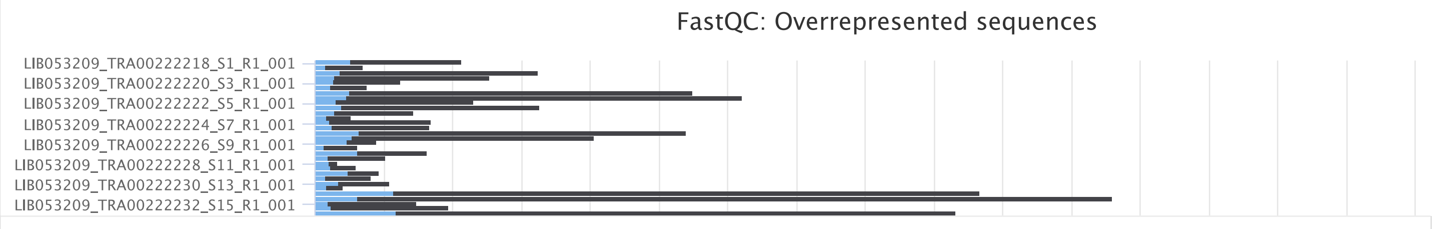

Adapter Content 61 35

Help

The cumulative percentage count of the proportion of your library which has seen each of the adapter sequences at each position.

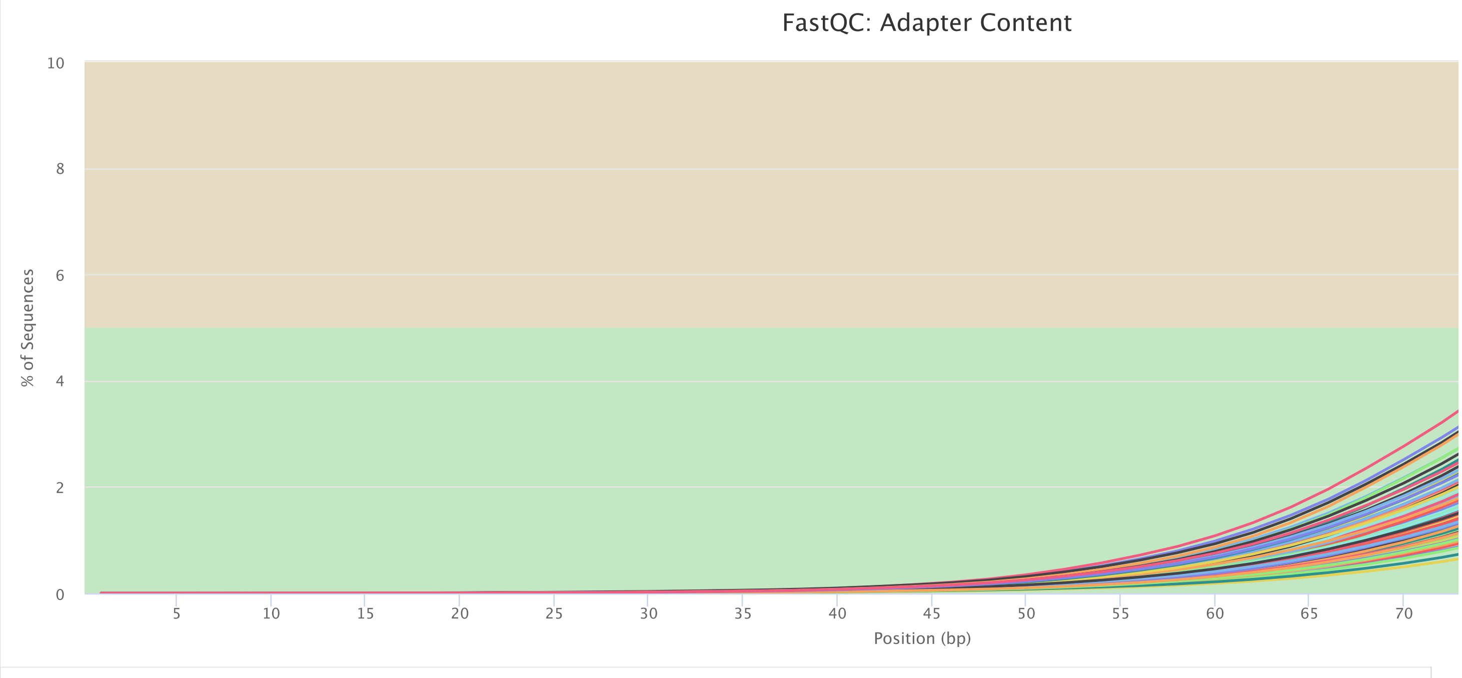

Status Checks

Help

Status for each FastQC section showing whether results seem entirely normal (green), slightly abnormal (orange) or very unusual (red).

Sort by highlight

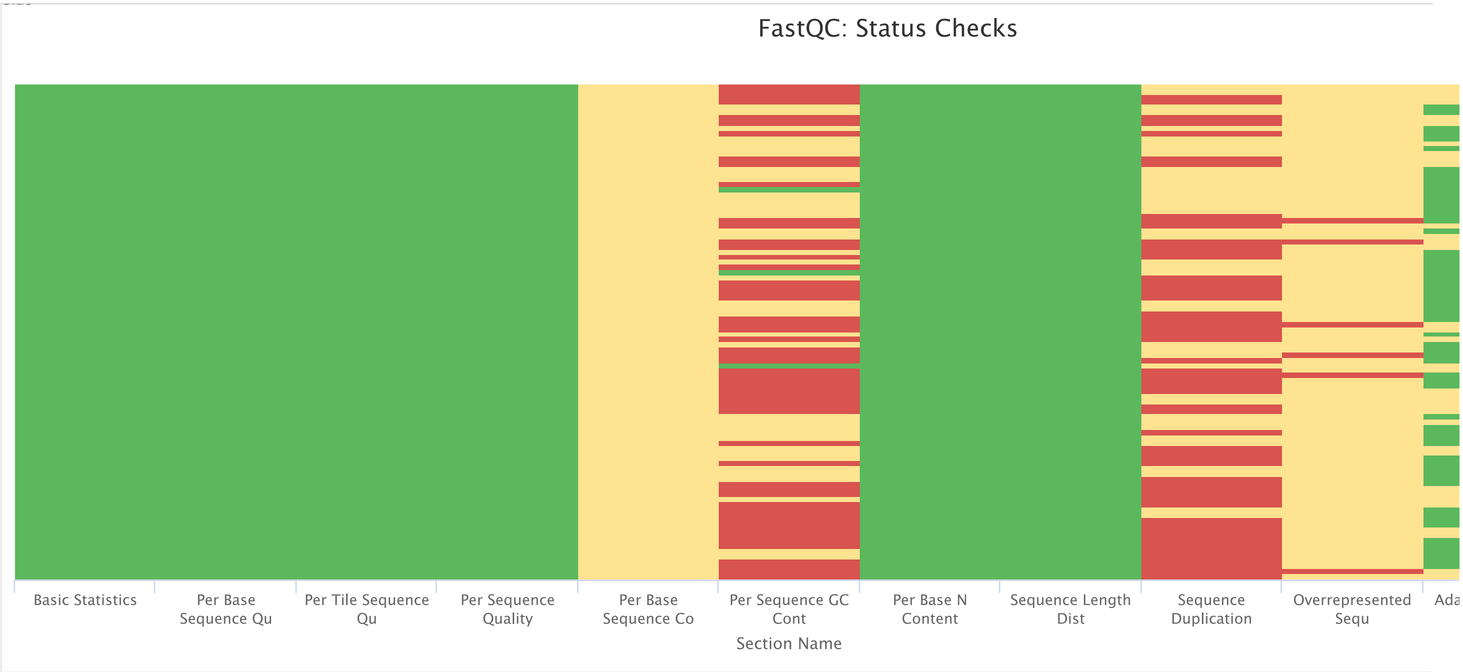

Supplement: Supplementary file 1 [file bioengineering-10-00527-s001.zip › Supplement S2/Supplement S2--MultiQC Reports.pdf]
