# Supplementary figures and images for: Responding to ACL Injury and its Treatments: Comparative Gene Expression between Articular Cartilage and Synovium
Source: Bioengineering (Basel). 2023 Apr 26;10(5):527. doi: 10.3390/bioengineering10050527 (PMC10215325; doi:10.3390/bioengineering10050527)

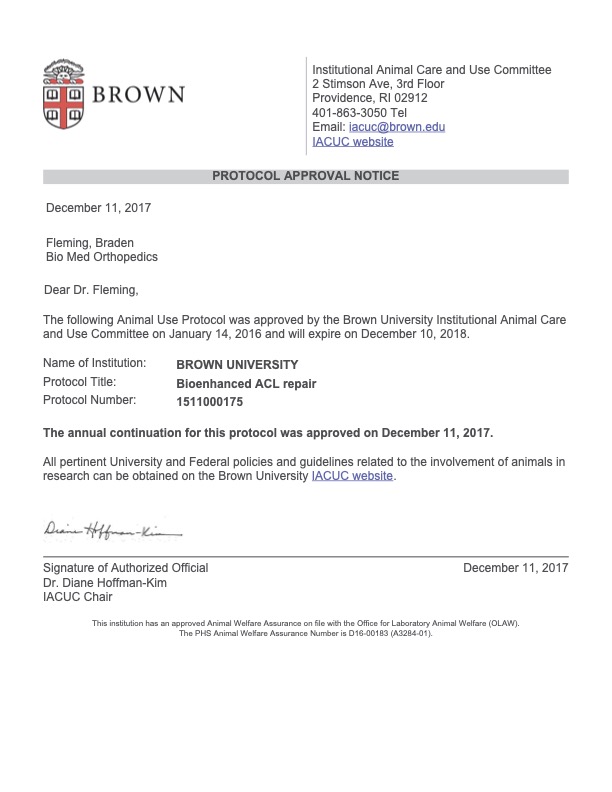

Supplement: Supplementary file 1 [file bioengineering-10-00527-s001.zip › Supplement S1/IACUC APPROVAL.jpg]

# Likely outlier—52W Synovium ACLT sample #12 (aka INJ52\_SM\_12 or INJ52\_12)

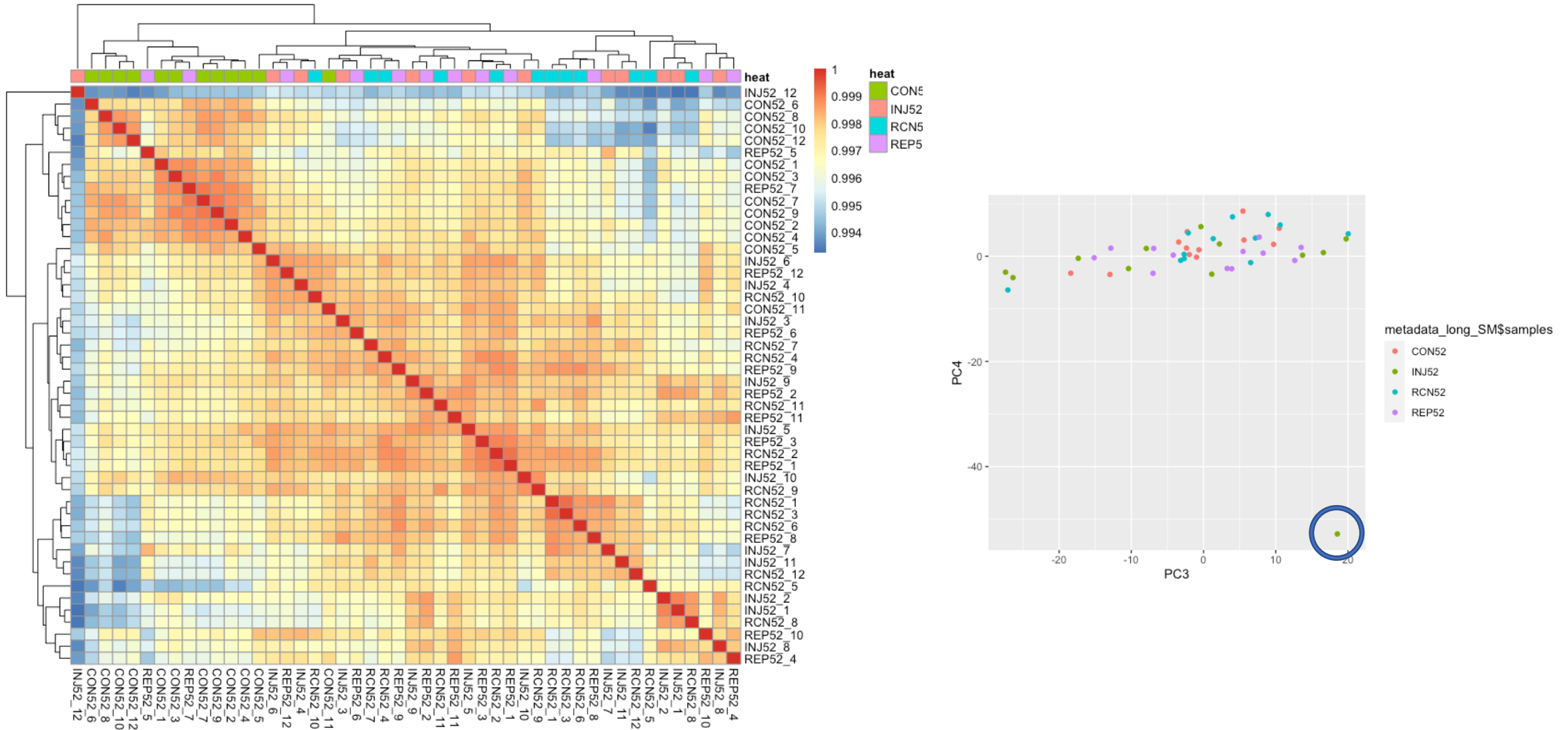

# After removing INJ52\_SM\_12

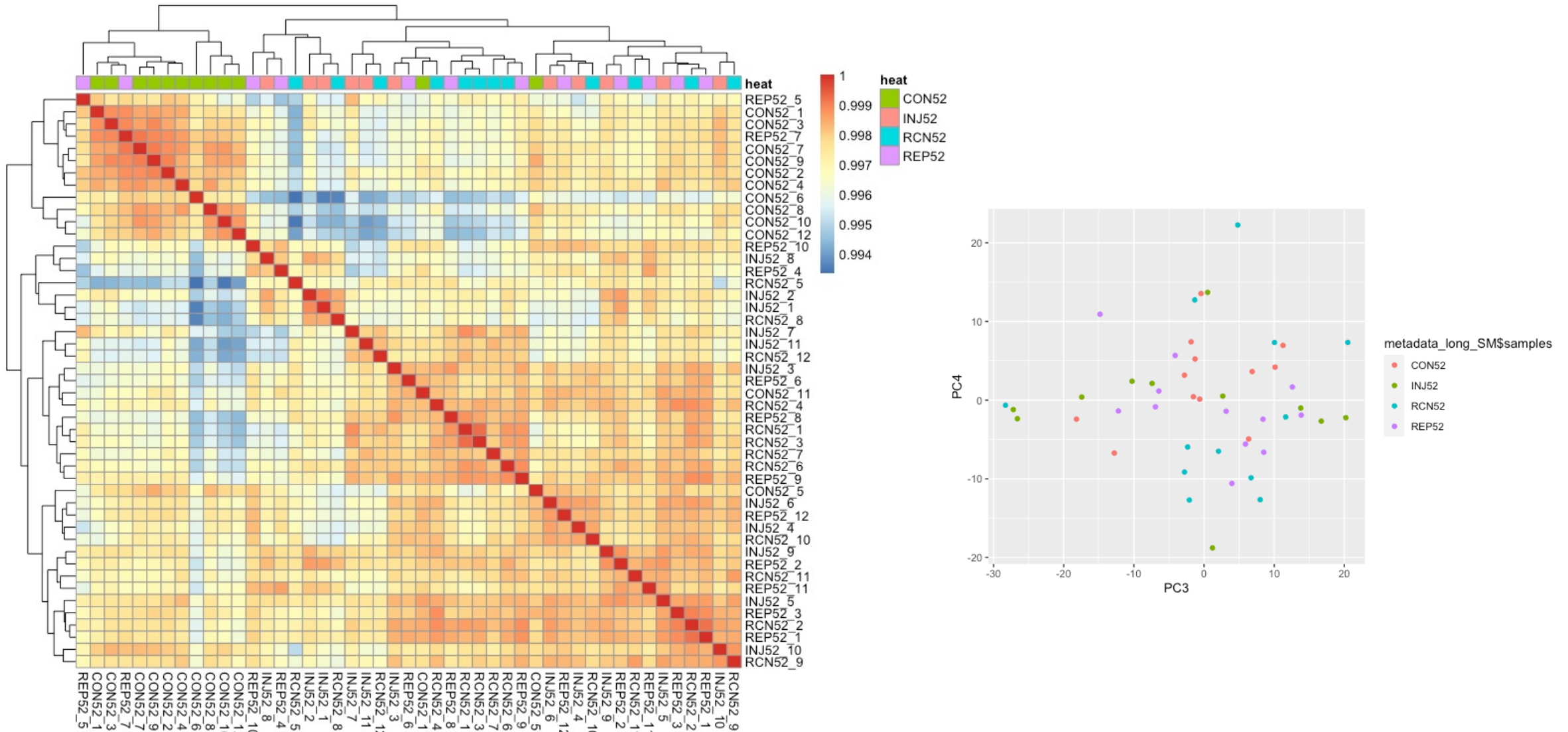

Supplement: Supplementary file 1 [file bioengineering-10-00527-s001.zip › Supplement S4/Supplement S4--PCA outliers.pdf]

## Slide 1
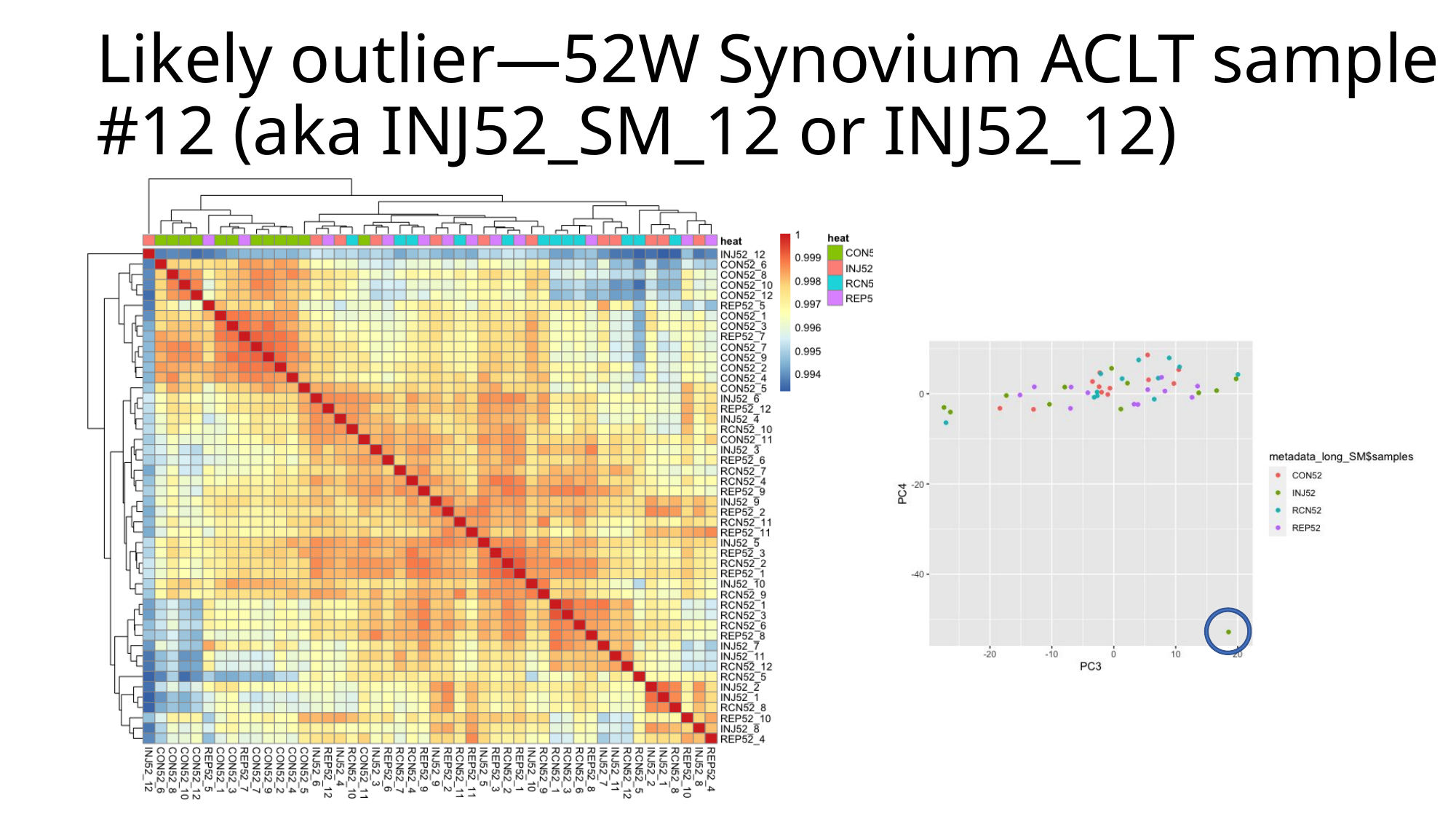

# Likely outlier—52W Synovium ACLT sample #12 (aka INJ52_SM_12 or INJ52_12)

## Slide 2
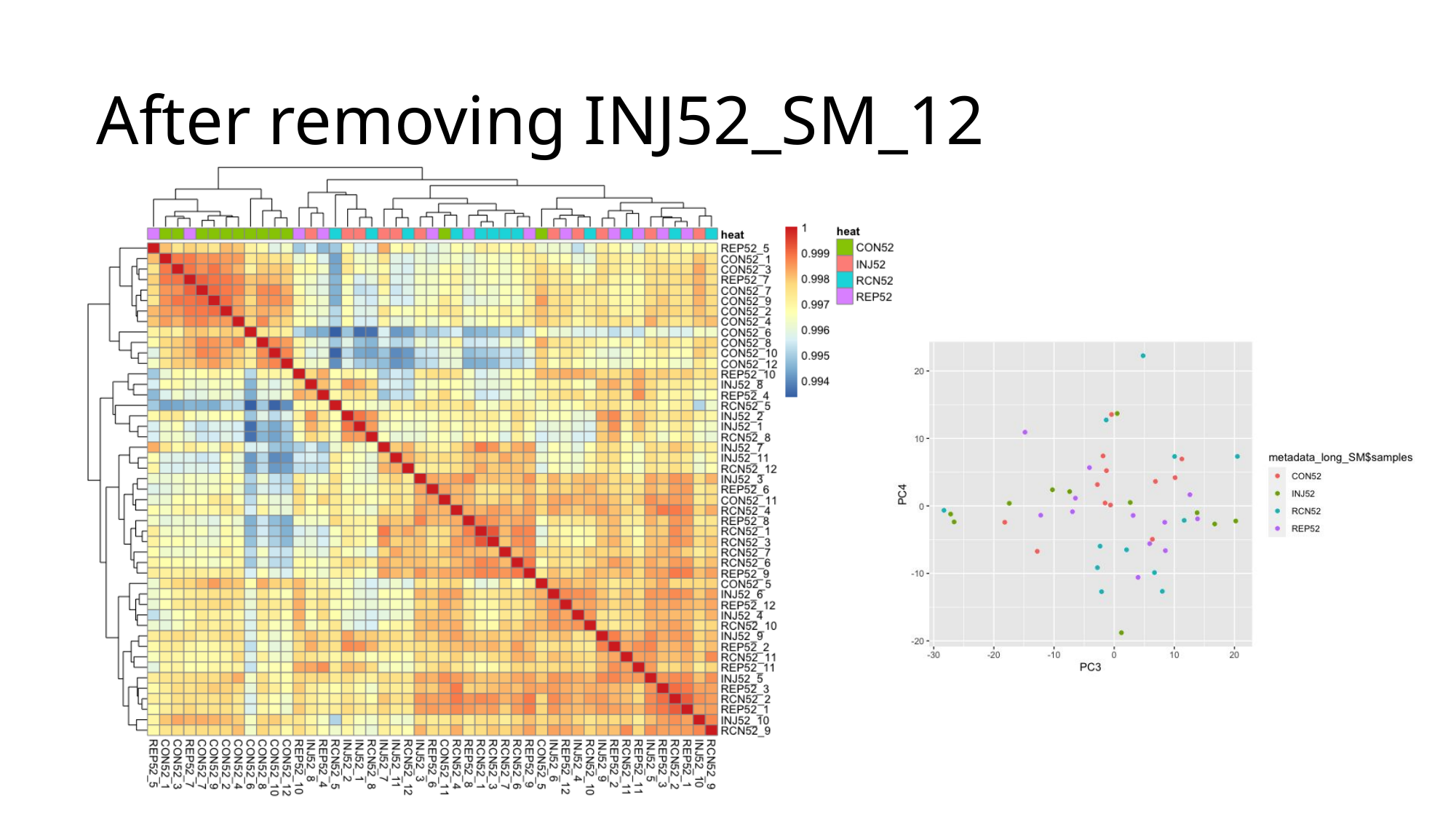

# After removing INJ52_SM_12

Supplement: Supplementary file 1 [file bioengineering-10-00527-s001.zip › Supplement S4/Supplement S4--PCA outliers.pptx]
